# Biomolecular mechanisms for signal differentiation

**DOI:** 10.1016/j.isci.2021.103462

**Published:** 2021-11-17

**Authors:** Emmanouil Alexis, Carolin C.M. Schulte, Luca Cardelli, Antonis Papachristodoulou

**Affiliations:** 1 Department of Engineering Science, University of Oxford, Oxford OX1 3PJ, UK; 2 Department of Plant Sciences, University of Oxford, Oxford OX1 3RB, UK; 3 Department of Computer Science, University of Oxford, Oxford OX1 3QD, UK

**Keywords:** Mathematical biosciences, Systems biology, Synthetic biology

## Abstract

Cells can sense temporal changes of molecular signals, allowing them to predict environmental variations and modulate their behavior. This paper elucidates biomolecular mechanisms of time derivative computation, facilitating the design of reliable synthetic differentiator devices for a variety of applications, ultimately expanding our understanding of cell behavior. In particular, we describe and analyze three alternative biomolecular topologies that are able to work as signal differentiators to input signals around their nominal operation. We propose strategies to preserve their performance even in the presence of high-frequency input signal components which are detrimental to the performance of most differentiators. We find that the core of the proposed topologies appears in natural regulatory networks and we further discuss their biological relevance. The simple structure of our designs makes them promising tools for realizing derivative control action in synthetic biology.

## Highlights

• Calculating the speed or higher derivatives of molecular signals has been a challenge
• Biomolecular topologies acting as signal differentiators are studied
• High performance and insensitivity to high-frequency input signals can be achieved
• Natural circuits and synthetic realizations for signal differentiation are described

## Graphical abstract

**Figure:**
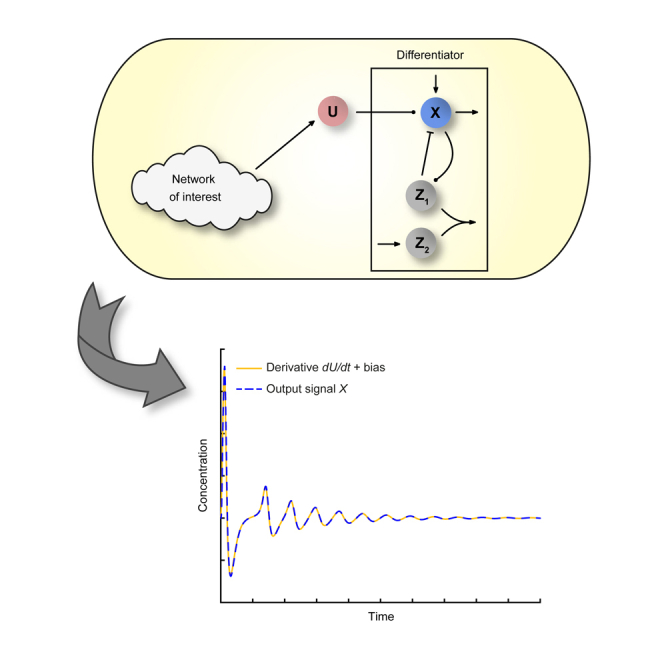

## Introduction

Measuring the speed at which a physical process evolves over time is of central importance to science and engineering. This can be done by computing the time derivative of the function describing the process. Several examples of cellular systems exhibiting derivative action indicate that calculating the rate of change of biological processes is essential in nature. The retina of our eyes, for instance, is one of the best-studied neural networks of the brain. Its response to changes in light intensity reveals typical characteristics of derivative action which stem from the interaction between cone and horizontal cells (Wilson, 1999; Åström and Murray, 2021). In microbiology, the chemotaxis signaling pathway in bacteria such as *Escherichia coli* involves computation of time derivatives: To navigate toward nutrients and away from toxins, bacteria are able to sample their environment as they move and convert spatial gradients into temporal ones (Alon, 2019; Shimizu et al., 2010; Iglesias and Devreotes, 2008; Barkai and Leibler, 1997; Block et al., 1983; Macnab and Koshland, 1972). Furthermore, in the context of cellular energy metabolism, *in silico* studies have revealed the role of creatine phosphate as a buffering species that allows for adaptation to a changing demand of adenosine triphosphate (ATP), thus exploiting the anticipatory action enabled by derivative control (Cloutier and Wellstead, 2010). This observation is a specific example of a broader class of biomolecular processes where the presence of rapid buffering proves to be equivalent to negative derivative feedback (Hancock et al., 2017).

In traditional engineering, differentiators refer to devices capable of applying time differentiation to an input stimulus, for example a mechanical or electrical signal. In the rapidly growing field of synthetic biology, the ability to build reliable biomolecular differentiators would offer considerable advantages (Steel et al., 2017; Del Vecchio et al., 2016; Lu et al., 2009). As an immediate application, such genetic circuits would be able to track the rate of change of the concentration of biomolecules, thus acting as speed biosensors. This is of interest when assessing uptake rates of certain molecules, such as uptake of pollutants into bacteria used for bioremediation (Chen and Wilson, 1997; Pieper and Reineke, 2000). They can also allow for advanced regulation strategies in the cellular environment by enabling the construction of more efficient bio-controllers, e.g., Proportional-Integral-Derivative (PID) control schemes, the workhorses of modern technological process control applications (Åström and Murray, 2021). In general, derivative control can enhance the stability of a feedback system and provide a smoother transient response.

Recent efforts in this rather underexplored research area include the design of a differentiator module consisting of linear input/output functions realized by specific processes of protein production (Halter et al., 2017; Halter et al., 2019). It has further been demonstrated that calculation of time derivatives is possible by using ultrasensitive topologies operating within a negative feedback loop (Samaniego et al., 2019), and a motif capable of computing positive and negative temporal gradients, which includes input delays and the idea of an incoherent feedforward loop, has been presented (Samaniego et al., 2020). With the aim of providing derivative action in PID control architectures, networks directly inspired by bacterial chemotaxis (Chevalier et al., 2019) or based on the so-called dual rail encoding have also been proposed (Whitby et al., 2021; Paulino et al., 2019). This approach enables the representation of both positive and negative signals via biomolecular species by decomposing a signal into two non-negative parts (Oishi and Klavins, 2011). Finally, a derivative controller tailored to gene expression is analyzed in (Modi et al., 2019), while in the PID architecture introduced in (Filo and Khammash, 2021), derivative control is carried out with inseparable connection to proportional and integral actions.

In this article, we introduce novel differentiator modules aiming to elucidate unexplored mechanisms that cells potentially exploit to achieve signal differentiation. In parallel, these motifs can pave the way for designing efficient and reliable synthetic signal differentiator devices in a cellular context. Notably, our motifs offer considerable ease of experimental implementation compared to some of the earlier discussed designs which are based on more “artificial” mechanisms such as dual-rail encoding. In addition, the motifs under consideration can function as independent, general-purpose differentiators, which may be a challenging task for other topologies, such as some control-oriented topologies showing derivative action. Moreover, under suitable tuning high accuracy of temporal derivative calculation for a wide range of molecular signals can be guaranteed.

**Figure 1:**
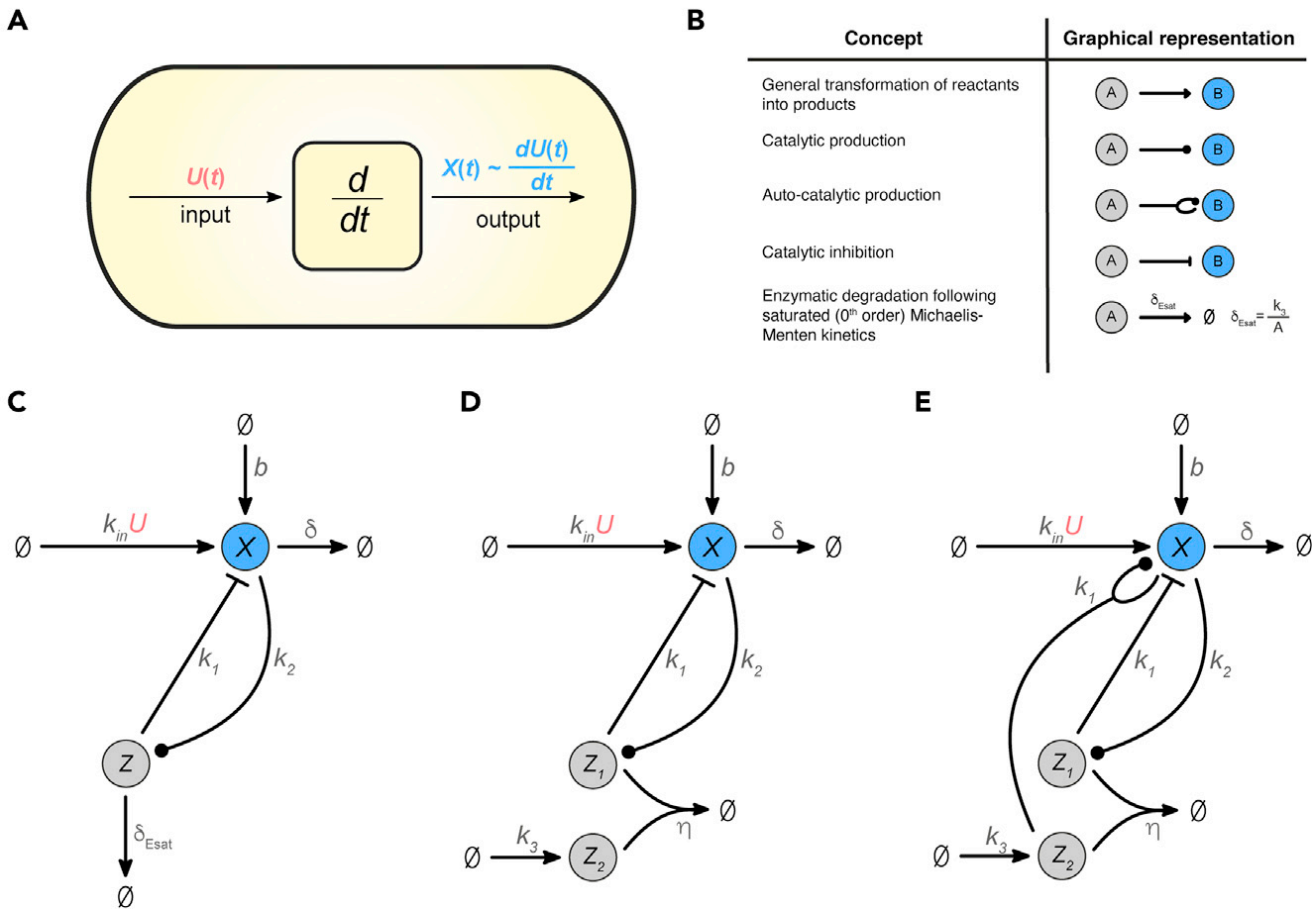
Biomolecular structures capable of signal differentiation (A) Schematic representation of the notion of signal differentiation carried out by a biomolecular device inside the cell. (B)Graphical representation of the biological concepts found in the signal differentiator motifs. To describe the different kind of biomolecular reactions the following notation is adopted: ( → ) means that the transformation of reactants into products only happens in the direction of the arrow. (—⋅) indicates that reactants enable product formation without being consumed. (—|) denotes inhibition of products by a reactant where the reactant is not consumed. In addition, the depicted concept of enzymatic degradation is further analyzed in STAR MethodsEquilibria and stability of biomolecular signal differentiators: Biomolecular Signal Differentiator-I. (C) Topology of Biomolecular Signal Differentiator - I or BioSD-I (Equation (1)). (D) Topology of Biomolecular Signal Differentiator - II or BioSD-II (Equation (2)). (E) Topology of Biomolecular Signal Differentiator - III or BioSD-III (Equation (3)).

Specifically, we present three biomolecular architectures capable of functioning as signal differentiators around their equilibria. We call them Biomolecular Signal Differentiators (BioSD). Each of these networks can be interpreted as a modular and tunable topology inside the cell that accepts a molecular signal as an input and produces an output signal proportional to the time derivative of the input signal (Figure 1A). The output corresponds to a biochemical species whose concentration can be measured. The proposed architectures provide simple blueprints for the design of synthetic biomolecular differentiators, but can also be interpreted as lenses through which derivative action in natural systems can be identified and studied.

We demonstrate the special characteristics and the performance trade-offs of the three BioSD architectures (BioSD-I, II, and III) via theoretical analyses and numerical simulations. We also discuss a major obstacle of both technological and biological differentiators, namely amplification of undesired high-frequency components of the input signal, and propose strategies to overcome this obstacle. Finally, we show the occurrence of one of the BioSD topologies in natural regulatory networks involved in bacterial adaptation to stress conditions and present potential synthetic implementations for all three topologies, highlighting the biological relevance of our designs.

## Results

### Biological structure

We begin by presenting the molecular interactions in the BioSD circuits as chemical reaction networks (CRNs). These circuits represent three alternative topological entities which, under certain assumptions, realize the same concept of signal differentiation. In the analysis that follows, the input and output signals of the differentiators are generally treated as biomolecular species, namely *U* and *X* respectively. Nevertheless, an input signal may also refer to different concepts such as light, temperature or pH.

Figure 1C illustrates the first architecture, BioSD-I, which consists of the following reactions:

(Equation 1) $$\begin{array}{c} \emptyset \overset{k_{in}U}{\to }X,\;\emptyset \overset{b}{\to }X,\;X\overset{k_{2}}{\to }X+Z\\ X+Z\overset{k_{1}}{\to }Z,\;X\overset{\delta }{\to }\emptyset ,\;Z\overset{\delta_{Esat}}{\to }\emptyset \end{array}$$

Here, the production of output species *X* depends on two reactions. One of them has a constant rate while the other occurs at a rate proportional to the concentration of input species *U*. It is convenient to represent such processes via reactions of the form $\emptyset \overset{r}{\to }X$, where *r* can be a constant or a time-varying quantity, e.g., biomolecular concentration. This allows us to describe general concepts of production without the need to specify their impact on the reactants involved. Furthermore, *X* also catalyzes the formation of species *Z* which, in turn, inhibits *X*. Note that the process of inhibition is interpreted as catalysis of degradation. Finally, the removal rate of *X* is proportional to its concentration (first-order decay) while, as indicated by the notation $\delta_{Esat}$ (defined in Figure 1B), *Z* adheres to a constant rate of decay (0th-order decay). The latter behavior is attained through enzyme-catalyzed degradation of *Z* where the enzyme is operating at saturating substrate levels (for more details see STAR Methods Equilibria and stability of biomolecular signal differentiators: biomolecular signal differentiator-I).

In the second architecture, BioSD-II (Figure 1D), the formation process of output species *X* is the same as in BioSD-I, while $Z_{1}$, the production of which is facilitated by *X*, and $Z_{2}$ annihilate each other. $Z_{1}$inhibits *X* which decays in the same way as in BioSD-I. The reactions that form the corresponding CRN are:

(Equation 2) $$\begin{array}{c} \emptyset \overset{k_{in}U}{\to }X,\;\emptyset \overset{b}{\to }X,\;X\overset{k_{2}}{\to }X+Z_{1}\\ X+Z_{1}\overset{k_{1}}{\to }Z_{1},\;\emptyset \overset{k_{3}}{\to }Z_{2},\;Z_{1}+Z_{2}\overset{\eta }{\to }\emptyset ,\;X\overset{\delta }{\to }\emptyset \end{array}$$

Finally, Figure 1E shows the third topology, BioSD-III, which is described by the reactions:

(Equation 3) $$\begin{array}{l} \emptyset \overset{k_{in}U}{\to }X,\;\emptyset \overset{b}{\to }X,\;X\overset{k_{2}}{\to }X+Z_{1},\;X+Z_{1}\overset{k_{1}}{\to }Z_{1}\\ \emptyset \overset{k_{3}}{\to }Z_{2},\;X+Z_{2}\overset{k_{1}}{\to }X+X+Z_{2},\;Z_{1}+Z_{2}\overset{\eta }{\to }\emptyset ,\;X\overset{\delta }{\to }\emptyset \end{array}$$

This CRN includes an autocatalytic-like reaction: *X* is able to produce more of itself in the presence of $Z_{2}$. The rest of its structure is identical to the CRN of BioSD-II.

### Mathematical description

We now derive the dynamics of the proposed BioSD networks using the law of mass action (Del Vecchio and Murray, 2015) unless otherwise stated, adopting the same order of presentation as in the preceding section.

BioSD-I (CRN given by Equation (1)) can be described by the following system of Ordinary Differential Equations (ODEs):

(Equation 4a) $$\dot{X}=k_{in}U+b-k_{1}XZ-\delta X$$

(Equation 4b) $$\dot{Z}=k_{2}X-k_{3}$$

Note that the enzymatic degradation of *Z* is assumed to follow saturated (0th-order) Michaelis-Menten kinetics, as previously discussed.

Next, from the CRN given by Equation (2) we obtain the following ODE model for BioSD-II:

(Equation 5a) $$\dot{X}=k_{in}U+b-k_{1}XZ_{1}-\delta X$$

(Equation 5b) $${\dot{Z}}_{1}=k_{2}X-\eta Z_{1}Z_{2}$$

(Equation 5c) $${\dot{Z}}_{2}=k_{3}-\eta Z_{1}Z_{2}$$

For the last circuit, BioSD-III, the CRN given by Equation (3) can be modeled using the following ODEs:

(Equation 6a) $$\dot{X}=k_{in}U+b-k_{1}XZ_{1}+k_{1}XZ_{2}-\delta X$$

(Equation 6b) $${\dot{Z}}_{1}=k_{2}X-\eta Z_{1}Z_{2}$$

(Equation 6c) $${\dot{Z}}_{2}=k_{3}-\eta Z_{1}Z_{2}$$

By assuming a constant input $U^{\text{∗}}$ and setting the derivatives to zero, we can show that each of the BioSD network models has a unique equilibrium. In addition, we can prove through linearization that the equilibrium is locally exponentially stable (a detailed analysis can be found in STAR Methods Equilibria and stability of biomolecular signal differentiators). Near their steady-states, the circuits are able to exhibit derivative action, as shown in the next section. Furthermore, for the purpose of this study we assume that the parameter *η* in BioSD-II is sufficiently large which can lead to a practically insignificant concentration of species $Z_{2}$ (more details can be found in STAR Methods The notion of strong rate of annihilation between Z1, Z2 (large η) in biomolecular signal differentiator-II). This constraint does not have to hold for BioSD-III, which includes the same annihilation reaction. Finally, Equations (5b) and (5c) indicate that in case ${\dot{Z}}_{2}\approx 0$, the removal rate of $Z_{1}$ is roughly constant and equal to $k_{3}$, similar to the 0th-order removal of *Z* in BioSD-I.

### Achieving biological signal differentiation

In order for the proposed biomolecular modules to work as signal differentiators, we desire for their output *X* to be proportional to the derivative of their input *U*. This immediately raises the following challenge: Both *U* and *X* refer to biomolecular species concentrations and, by extension, represent non-negative signals. However, in the general case, the derivative of a nonnegative signal can take negative values and, as a result, *X* would need to go below zero. Thus, it could be argued that *X* is unable to express the rate of change of an arbitrary input signal. An obvious way to overcome this obstacle is to add a bias to the computed derivative. As we demonstrate here, the perfect candidate for realizing this bias is the steady state of *X* around which derivative action can be achieved.

We are interested in the local behavior of the BioSD networks and, therefore, consider input stimuli that do not force them to operate far away from their equilibrium. Subsequently, we assume that every input signal can be described as:

(Equation 7) $$U=U^{\text{∗}}+U^{TV}$$

where $U^{\text{∗}}$ is constant while $U^{TV}$ is time-varying. Here, we focus on Fourier transformable signals which is typically the case for physical signals in practical applications (for more details see STAR Methods Signals under consideration).

By linearizing and applying appropriate transformations, we can show that the dynamics of the output of any of the three BioSD topologies presented in the previous section can be approximated by the following non-dimensional second - order differential equation (see STAR Methods Behavior analysis of biomolecular signal differentiators):

(Equation 8) $$\varepsilon {\ddot{x}}_{n}+\varepsilon {\dot{x}}_{n}+x_{n}={\dot{u}}_{n}$$

where$x_{n}$ and $u_{n}$ refer to the output and input, respectively and:

(Equation 9) $$\varepsilon =\frac{k_{2}^{2}}{k_{1}k_{3}^{3}}{\left(k_{in}U^{\text{∗}}+b\right)}^{2}$$

Equation (8) represents a signal differentiator accompanied with some filtering action. Indeed, the input/output relation in the Laplace domain can be described by the following transfer function (Oppenheim et al., 1996):

(Equation 10) $${\tilde{\text{Δ}}}_{BSD}\left(s\right)=\frac{{\tilde{X}}_{n}\left(s\right)}{{\tilde{U}}_{n}\left(s\right)}=\frac{s}{\varepsilon \left(s^{2}+s\right)+1}$$

where${\tilde{X}}_{n}\left(s\right)$ and ${\tilde{U}}_{n}\left(s\right)$ are the Laplace transform of the output $x_{n}$ and input $u_{n}$, respectively and *s* is the Laplace variable (complex frequency). As can be seen from Equation (10), a BioSD network is the series combination of an ideal differentiator and a second-order low pass filter (Samoilov et al., 2002). Therefore, for a given positive *ε*, the accuracy of signal differentiation depends on the frequency spectrum of the input signal or, in other words, the range of frequencies contained by it (see STAR Methods Signals under consideration). Accompanying a differentiator with a low-pass filter is a widely used strategy in traditional engineering in order to deal with high-frequency input noise (this topic is analyzed in Response to input signals corrupted by high-frequency noise and A structural addition for enhanced performance).

To gain a deeper insight, we calculate the Fourier transform (Oppenheim et al., 1996) of the output:

(Equation 11) $${\tilde{X}}_{n}\left(j\omega \right)={\tilde{\text{Δ}}}_{BSD}\left(j\omega \right){\tilde{U}}_{n}\left(j\omega \right)$$

where *ω* represents the frequency, *j* is the imaginary unit number ($j=\sqrt{-1}$) and ${\tilde{X}}_{n}\left(j\omega \right)$, ${\tilde{U}}_{n}\left(j\omega \right)$ are the Fourier transform of the output $x_{n}$ and input $u_{n}$, respectively. Furthermore, ${\tilde{\text{Δ}}}_{BSD}\left(j\omega \right)$ is the Fourier transform of the system's impulse response, also known as the frequency response of the system. (ibid.). Since we have a linear, asymptotically stable, system we can compute the latter Fourier transform from Equation (10) by setting s=jω. Thus, we have:

(Equation 12) $${\tilde{X}}_{n}\left(j\omega \right)=\frac{j\omega }{\varepsilon \left(-\omega^{2}+j\omega \right)+1}{\tilde{U}}_{n}\left(j\omega \right)$$

The operation of (ideal) differentiation in the frequency domain is defined as:

(Equation 13) $${\tilde{X}}_{nd}\left(j\omega \right)=j\omega {\tilde{U}}_{n}\left(j\omega \right)$$

To compare the output of an ideal differentiator to the one of a BioSD device, we introduce the following performance metric:

(Equation 14) $$\tilde{\text{Λ}}\left(j\omega \right)=\frac{{\tilde{X}}_{n}\left(j\omega \right)}{{\tilde{X}}_{nd}\left(j\omega \right)}=\frac{1}{\varepsilon \left(-\omega^{2}+j\omega \right)+1}$$

Using the magnitude-phase representation of Equation (14) we get:

(Equation 15) $$\left|\tilde{\text{Λ}}\left(j\omega \right)\right|=\frac{1}{\sqrt{\varepsilon^{2}\omega^{2}+{\left(1-\varepsilon \omega^{2}\right)}^{2}}}$$

and

$$∠\tilde{\text{Λ}}\left(j\omega \right)=\text{arctan}\left(\frac{-\varepsilon \omega }{1-\varepsilon \omega^{2}}\right)$$

**Figure 2:**
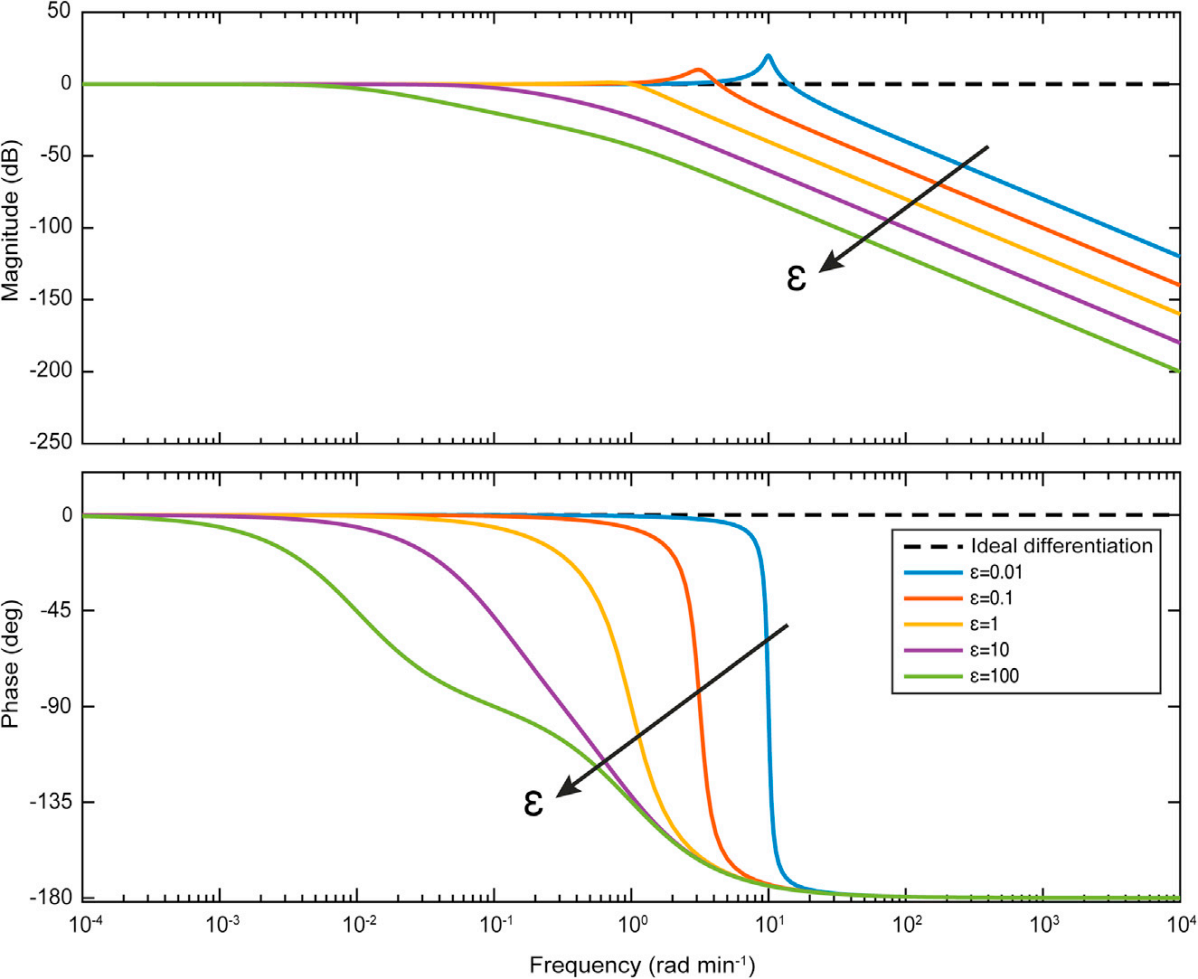
A performance metric for Biomolecular Signal Differentiators in the frequency domain Bode plot of the metric given by Equation (14). Different colors represent the magnitude and the phase of the corresponding transfer function for different values of *ε*. The case of ideal differentiation corresponds to ε=0 and the direction in which the latter increases indicated by an arrow.

Signal differentiation of high accuracy is carried out when $\tilde{\text{Λ}}\left(j\omega \right)$ is close to $1∠0^{\circ }$. As shown in Figure 2, there is a “low-frequency” range where this is true, but as *ε* decreases the aforementioned range expands toward “higher frequencies”. In the time domain this entails that for a given positive *ε*, a BioSD device can work as an accurate signal differentiator for sufficiently slow input signals and, in that case, the BioSD output can be approximated by (see STAR Methods Behavior analysis of biomolecular signal differentiators):

(Equation 16) $$X=\frac{k_{in}}{k_{1}k_{3}}\dot{U}+\frac{k_{3}}{k_{2}}$$

There is a family of input signals for which the BioSD topologies are able to provide accurate differentiation regardless of the exact value of *ε* (see STAR Methods Behavior analysis of biomolecular signal differentiators). More specifically, this holds for input signals for which the term $U^{TV}$ in Equation (7) is of the form:

(Equation 17) $$U^{TV}=\xi_{1}e^{-\xi_{3}t}+\xi_{2}t,$$

where $\xi_{1}$, $\xi_{2}$ are arbitrary constants and $\xi_{3}=\frac{k_{2}}{k_{3}}\left(k_{in}U^{\text{∗}}+b\right)$. If $\xi_{2}$ is not zero which implies linear growth over time, we assume that the above holds as long as the system stays near its equilibrium. This means that the term $\xi_{2}t$ is sufficiently small. Indeed, several biological processes can generate (bounded) signals some part of which can be viewed as linear growth (Del Vecchio and Murray, 2015). We study such a scenario in Sensing the response speed of biomolecular networks.

As Equation (8) states, the response of a BioSD network, is given as the solution of a second-order non-homogeneous differential equation with constant coefficients where the forcing function is ${\dot{u}}_{n}$. The response can therefore be seen as the sum of two terms: a “transient” term which highly depends on the initial conditions and dies out with time; and a “steady-state” term which, under the conditions discussed above, can approximate the derivative of the input signal (Zill, 2012). Therefore, for input signals applied for a sufficiently long time, the BioSD output practically coincides with the latter since the effect of the former is negligible. However, this may not be always the case for short duration input signals where any undesired initial transient phenomena can greatly compromise the accuracy of the differentiator output.

From Equation (16), we can see that the BioSD modules use the biomolecular concentration $\frac{k_{3}}{k_{2}}$ as a bias. Around this point they can operate as signal differentiators, producing an output signal component which is proportional to the derivative of the input. The bias therefore depends only on two parameters which, ideally, can be adjusted as desired. This provides us with the freedom of choosing any (fixed) concentration of *X* as a bias, which will remain unchanged regardless of the rest of the model parameters, the input stimulus, or potential constant disturbances on the output. To appreciate this further, we recall the production reaction for *X* with constant rate *b*, which is included in each of the proposed CRNs. Besides its role as a structural requirement, this production reaction can also represent an external constant disturbance applied on *X*; this, however, does not affect the zero-level we choose for our measurements. Once the concentration of *X* reaches this level, it will stay there until an input excitation appears and it will come back once the excitation stops. Hence, the previously mentioned fixed concentration can also be seen as a “rest position” for the differentiators.

The feature just described is of key importance and stems mainly from the following two sources: the stability that characterizes BioSDs and the fact that the steady-state of the output coincides with the aforementioned zero-level concentration. The latter is achieved due to integration carried out by the ‘memory’ function which is realized via species *Z* within BioSD-I and the quantity $Z_{1}-Z_{2}$ within BioSD-II, III.

### Tunability and accuracy

It is convenient for the circuit designer who aims to implement the BioSD topologies to be able to choose the parameter values and ensure that the resulting differentiators meet the expected performance requirements. Nonetheless, there may be cases where the number of system parameters that can be suitably tuned is limited, for instance due to constraints related to the cellular processes involved in the circuits under investigation. Even in this case, the architecture of our circuits allows for some tunability as long as the designer can choose some crucial parameters.

Consider for example the extreme scenario where only one of the model parameters can be regulated. If this parameter is $k_{3}$, then, according to Equation (16), its appropriate tuning may result in an acceptable gain by which the output signal is multiplied (output gain) and bias based on which this signal is measured. At the same time, Equation (9) reveals that (contrary to other parameters) a small change in $k_{3}$ can affect *ε* significantly since the latter is inversely proportional to the cube of $k_{3}$.

It immediately emerges from the above that the way we tune the BioSD networks defines the level of accuracy regarding their derivative action. Indeed, *ε* is subject to almost all parameter rates in these networks and, as pointed out in the previous section, the value of *ε* defines the range of frequencies over which BioSDs can accurately compute the rate of change of a biological signal.

### Sensing the response speed of biomolecular networks

We now demonstrate through an example the ability of BioSD modules to compute the temporal derivative of biological signals. At the same time, we highlight one of their potential applications discussed above, namely as rate-of-change detectors or speed biosensors.

**Figure 3:**
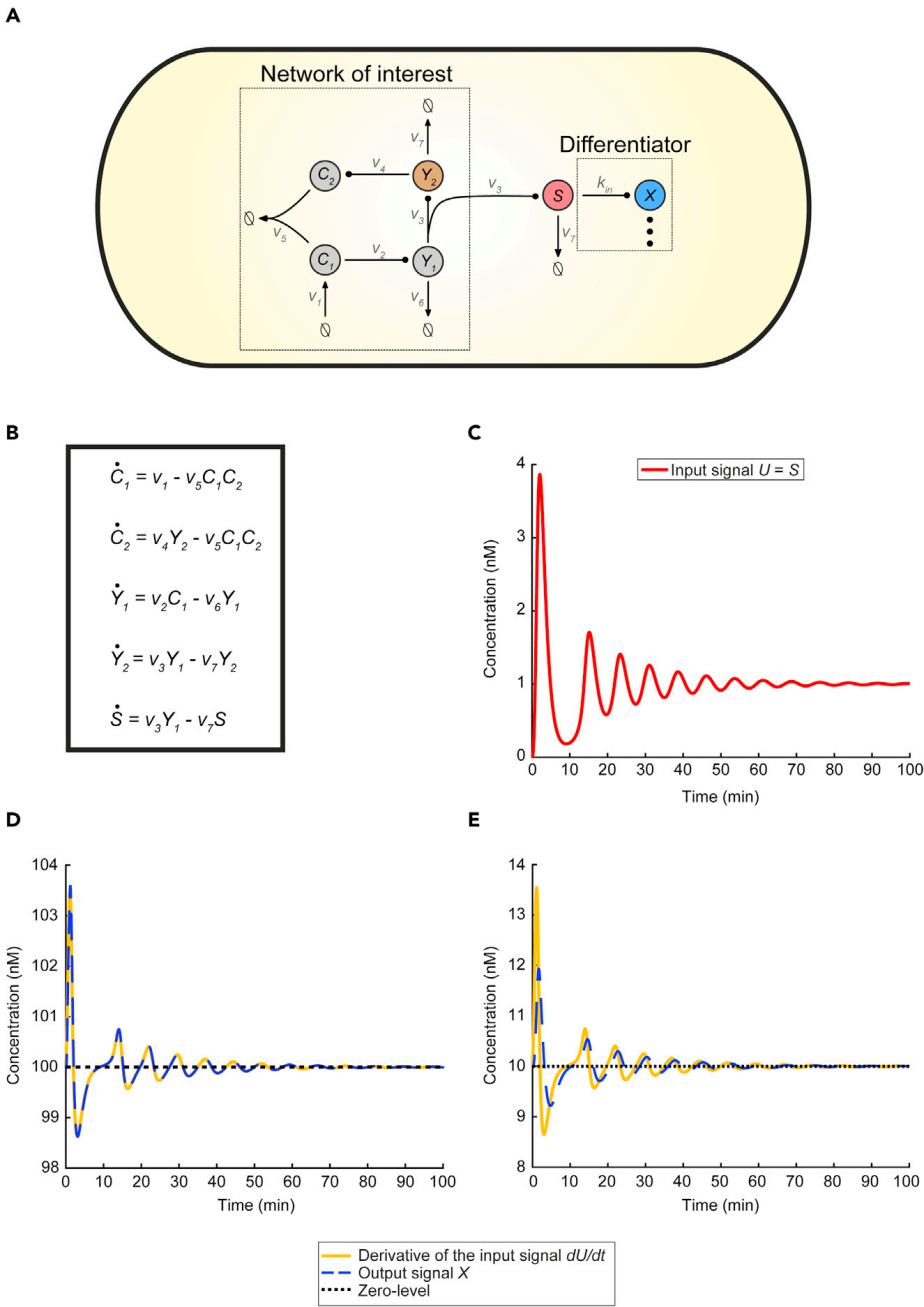
Sensing the rate-of-change of the output of a synthetic regulatory biomolecular network through a Biomolecular Signal Differentiator (A) Schematic of CRN (18) (network of interest) accompanied by a BioSD device (differentiator) which measures the speed of the output, $Y_{2}$ of the network via the sensing mechanism in Equation (20). We adopt the same arrow notation as in Figure 1 while the symbol (⋮) represents any of the three BioSD devices. (B) ODE model capturing the dynamics of the topology given by Equations (18) and (20). As anticipated, the behavior of species $Y_{2}$ and *S* is described by the same equation. (C) Input *U* of the differentiator coincides with species *S* and results from the simulation of the ODE model depicted in (B) with the following parameters: $\nu_{1}=2$ nM min^−1^, $\nu_{2}=$$\nu_{4}=2$ min^−1^, $\nu_{3}=4$ min^−1^, $\nu_{5}=12$ nM^−1^ min^−1^ , $\nu_{6}=$$\nu_{7}=1$ min^−1^. (D) Simulation of BioSD-I (Equations (4a) and (4b)) response to the input shown in (C) using the following parameters: $k_{in}=100$ min^−1^, $k_{3}=$b=100 nM min^−1^, $k_{1}=1$ nM^−1^ min^−1^, $k_{2}=1$ min^−1^, δ=0.5 min^−1^. Equation (9) therefore yields ε=0.01. As can be seen, the output, *X*, of the differentiator is an accurate replica of the derivative of input *U*. (E) The simulation in (D) is repeated after replacing the value of both $k_{in}$ and $k_{3}$ with 10. Equation (9) therefore yields ε=10. Although the output, *X*, of the differentiator remains close to the derivative of input *U*, there is some loss of accuracy compared to (D). The respective simulations regarding BioSD-II and BioSD-III are presented in Figure S1. As expected, their responses are identical to those of BioSD-I.

We consider the antithetic motif (Figure 3) (Briat, Gupta, and Khammash, 2016, 2018; Chevalier et al., 2019; Olsman et al., 2019a, Olsman et al., 2019b; Olsman and Forni, 2020; Baetica et al., 2020):

(Equation 18) $$\begin{array}{c} \emptyset \overset{\nu_{1}}{\to }C_{1},\;C_{1}\overset{\nu_{2}}{\to }C_{1}+Y_{1},\;Y_{1}\overset{\nu_{3}}{\to }Y_{1}+Y_{2}\\ Y_{2}\overset{\nu_{4}}{\to }Y_{2}+C_{2},\;C_{1}+C_{2}\overset{\nu_{5}}{\to }\emptyset ,\;Y_{1}\overset{\nu_{6}}{\to }\emptyset ,\;Y_{2}\overset{\nu_{7}}{\to }\emptyset \end{array}$$

Species $Y_{1}$, $Y_{2}$ represent an arbitrary biological process whose output, $Y_{2}$, can be robustly steered toward a desired value $\left(\frac{\nu_{1}}{\nu_{4}}\right)$. This is feasible through the feedback integral control which is implemented via species $C_{1}$, $C_{2}$, thus achieving robust perfect adaptation. Depending on the parameter rates, the dynamics of the above architecture can be either stable or unstable. Nonetheless, even in a stable system, the species of interest, $Y_{2}$, sometimes displays a long-lasting transient response with damped oscillations before it settles to a steady-state. This provides an opportunity to assess the ability of the BioSD networks to calculate the speed at which these oscillations evolve.

In order for a BioSD device to function as a biosensor for the CRN given by (Equation 18), a suitable interconnection between these circuits is required while preserving the modularity of the two networks and avoiding any loading problems, i.e., effects of retroactivity (Del Vecchio and Murray, 2015; Del Vecchio et al., 2008, 2016). One way to accomplish this is through the reaction:

(Equation 19) $$Y_{2}\overset{k_{in}}{\to }Y_{2}+X$$

where$Y_{2}$ plays the role of the input species *U* without being consumed. Alternatively, in case the nature of $Y_{2}$ prevents it from directly producing *X*, we can use a separate sensory species *S* which is capable of participating in the formation of *X*. In particular, we assume that *S* is co-expressed with and decays at the same rate as $Y_{2}$, i.e.:

(Equation 20) $$Y_{1}\overset{\nu_{3}}{\to }Y_{1}+Y_{2}+S,\;S\overset{k_{in}}{\to }S+X,\;S\overset{\nu_{7}}{\to }\emptyset$$

Adopting the second interconnection as the most general one, we demonstrate in Figure 3 that the rate of change of the concentration of $Y_{2}$ can be accurately represented by the output of the BioSD networks. We also demonstrate that, for a given input signal, there exist sufficiently large values of *ε* for which the BioSD performance may not be satisfactory due to some loss of accuracy (discussed in Achieving biological signal differentiation).

**Figure 4:**
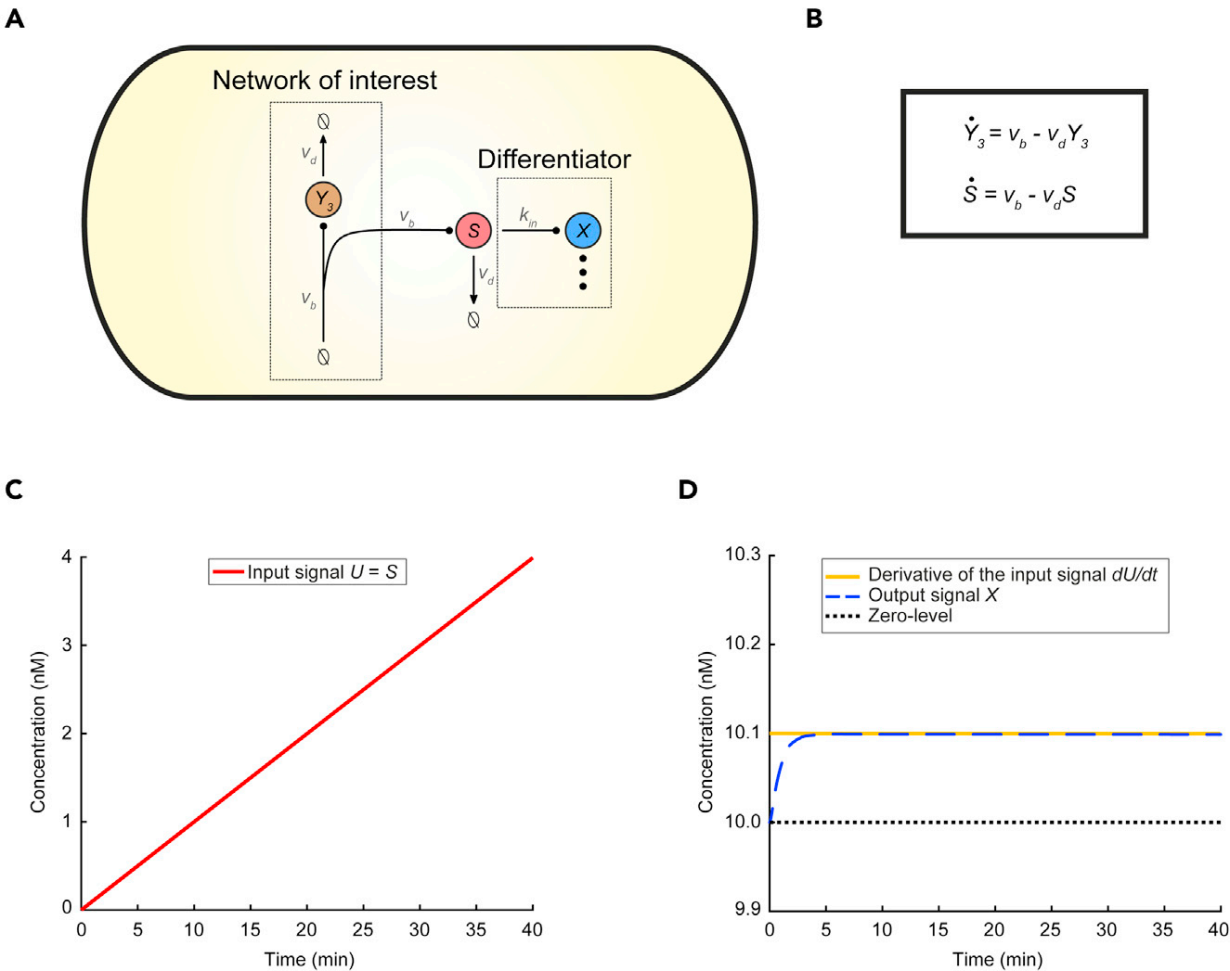
Sensing the rate-of-change of a production - removal biomolecular process through a Biomolecular Signal Differentiator (A) Schematic of CRN (21) (network of interest) accompanied by a BioSD device (differentiator), which measures the speed of the output of the network ($Y_{3}$) via the sensing mechanism in Equation (20). We adopt the same arrow notation as in Figure 1 while the symbol (⋮) represents any of the three BioSD devices. (B) ODE model capturing the dynamics of the topology given by Equations (20) and (21). As anticipated, the behavior of species $Y_{3}$ and *S* is described by the same equation. (C) Input *U* of the differentiator coincides with species *S* and results from the simulation of the ODE model depicted in Bwith the following parameter values: $\nu_{b}=0.1$ nM min^−1^, $\nu_{d}=0.001$ min^−1^. (D) Simulation of the BioSD-I (Equation (4a),(4b)) response to the input presented in (C) using the following parameters: $k_{in}=10$ min^−1^, $k_{3}=10$ nM min^−1^, b=100 nM min^−1^, $k_{1}=1$ nM^−1^ min^−1^, $k_{2}=1$ min^−1^, δ=0.5 min^−1^ (same as in Figure 3E, ε=10). The output, *X*, of the differentiator is now an accurate replica of the derivative of input *U*. The latter (shown in C) belongs to the class of signals defined by Equations (7) and (17). The respective simulations regarding BioSD-II and BioSD-III are presented in Figure S2. As expected, their responses are identical to those of BioSD-I.

We now replace the circuit described by (18) with the general production-removal process:

(Equation 21) $$\emptyset \overset{\nu_{b}}{\to }Y_{3},\;Y_{3}\overset{\nu_{d}}{\to }\emptyset$$

maintaining the same type of interconnection, as illustrated in Figure 4. Although the response of this process eventually converges to an equilibrium, for some period of time it practically increases linearly with time. Here, we focus on this linear regime of the response which is clearly aligned with Equation (17). Thus, as can be seen from Figure 4, BioSD networks are now able to provide accurate signal differentiation regardless of the high value of *ε* which, in the case of Figure 3, lead to a noticeable loss of accuracy.

### Response to input signals corrupted by high-frequency noise

Potentially the most important problem of differentiator devices is their sensitivity to high-frequency noise components which the applied input signal may contain (Åström and Murray, 2021). To this end, we consider an input signal with a time-varying component

(Equation 22) $$U^{TV}=\underset{\text{useful information}}{\underset{︸}{A_{u}\text{sin}\left(\omega_{u}t+\varphi_{u}\right)}}+\underset{\text{noise}}{\underset{︸}{A_{d}\text{sin}\left(\omega_{d}t+\varphi_{d}\right)}}$$

where the actual signal we want to differentiate–useful information–is accompanied by undesired fluctuations (noise) arising, for instance, from unintended cross-talk interactions (Del Vecchio and Murray, 2015). Note that although we model both the useful information and the noise as sinusoids, this is without loss of generality as they can be thought of as Fourier components of more general signals (see STAR Methods Signals under consideration). Assuming perfect differentiation, we get:

(Equation 23) $${\dot{U}}^{TV}=\underset{\text{derivative of useful information}}{\underset{︸}{\omega_{u}A_{u}\text{sin}\left(\omega_{u}t+\varphi_{u}+\frac{\pi }{2}\right)}}+\underset{\text{derivative of noise}}{\underset{︸}{\omega_{d}A_{d}\text{sin}\left(\omega_{d}t+\varphi_{d}+\frac{\pi }{2}\right)}}$$

Hence, even if the level of input corruption is low (e.g., $A_{d}$ is much smaller than $A_{u}$ - Equation (22)), the damage in the output of a perfect differentiator may be detrimental in case of a rapidly fluctuating noise signal ($\omega_{d}$ high). That is, $\omega_{d}A_{d}$ can be made arbitrarily large compared to $\omega_{u}A_{u}$ (Equation (23)) and, therefore, it is possible for the derivative of the useful signal to be completely drowned out by the derivative of some high frequency input noise. It is also apparent that the behavior of such an ideal differentiator module in the cellular environment is undesirable since it can lead to generation of greatly amplified output signals, which can be catastrophic.

**Figure 5:**
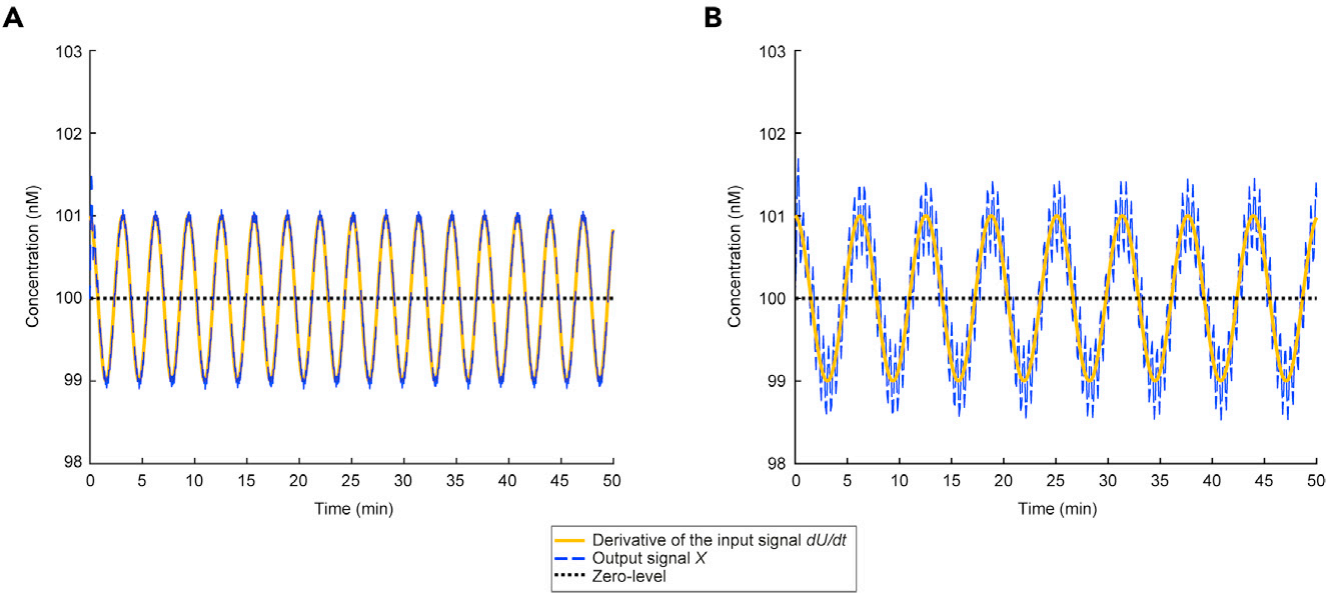
Response of Biomolecular Signal Differentiators to input signals with undesired high frequency components (A) Without loss of generality we select BioSD-I (Equations (4a) and (4b)) to plot: A simulated response to an input of the form given by Equations (7) and (22) using the following parameters: $U^{\text{∗}}=1.2$ nM, $A_{u}=1$ nM $\omega_{u}=1$ rad min^−1^, $A_{d}=0.2$ nM, $\omega_{d}=400$ rad min^−1^, $\varphi_{u}=\varphi_{d}=0$ rad, $k_{in}=100$ min^−1^, $k_{3}=$b=100 nM min^−1^, $k_{1}=1$ nM^−1^ min^−1^, $k_{2}=1$ min^−1^, δ=0.5 min^−1^. Equation (9) therefore yields ε=0.0484. Consequently, with respect to the input signal, the frequency of the undesired component (noise) is 400 times higher than that of the component of interest (useful information). It is evident that significant noise attenuation takes place and the accuracy of signal differentiation therefore remains very high. (B) The simulation in (A) is repeated after changing the value of $\omega_{d}$ to 50 which makes the noise 50 times faster compared to the useful information. As can be seen, there is a decrease in the accuracy level of signal differentiation since the input noise of this frequency cannot be filtered adequately. For demonstration purposes, in both (A) and (B) we have chosen a baseline (around of which derivative action is carried out) much larger than the amplitudes of the (ideal) derivatives regarding all the input stimuli. The useful information is represented by a signal component whose (ideal) derivative has an amplitude much smaller than the one of the (ideal) derivative of the noise. Consequently, the former can be drowned out by the latter if no noise attenuation is performed.

Interestingly, the BioSD topologies allow us to deal with this noise amplification by suitably adjusting *ε*. As already discussed, BioSDs possess a low-pass filtering property defined by *ε* (see Equation (10)). Although this may be viewed as an “imperfection” in terms of their signal differentiation ability, it turns out to be a saving feature of great significance. Recalling the performance metric given by Equation (10) which coincides with the frequency response of the embedded filter and the Bode plot of Figure 2, we can see that there is a range of high frequencies over which signal attenuation can be effectively performed (see also STAR Methods Behavior analysis of biomolecular signal differentiators and Figure S3). This implies that Equation (15) approaches zero. Moreover, as *ε* increases, this range expands toward lower frequencies. Nevertheless, between the aforementioned range and the low-frequency one corresponding to signal differentiation, we can detect the existence of a relatively narrow frequency band where BioSD circuits may not be able to differentiate or attenuate input signals with satisfactory accuracy. The characteristics described above are demonstrated in Figure 5.

### A structural addition for enhanced performance

**Figure 6:**
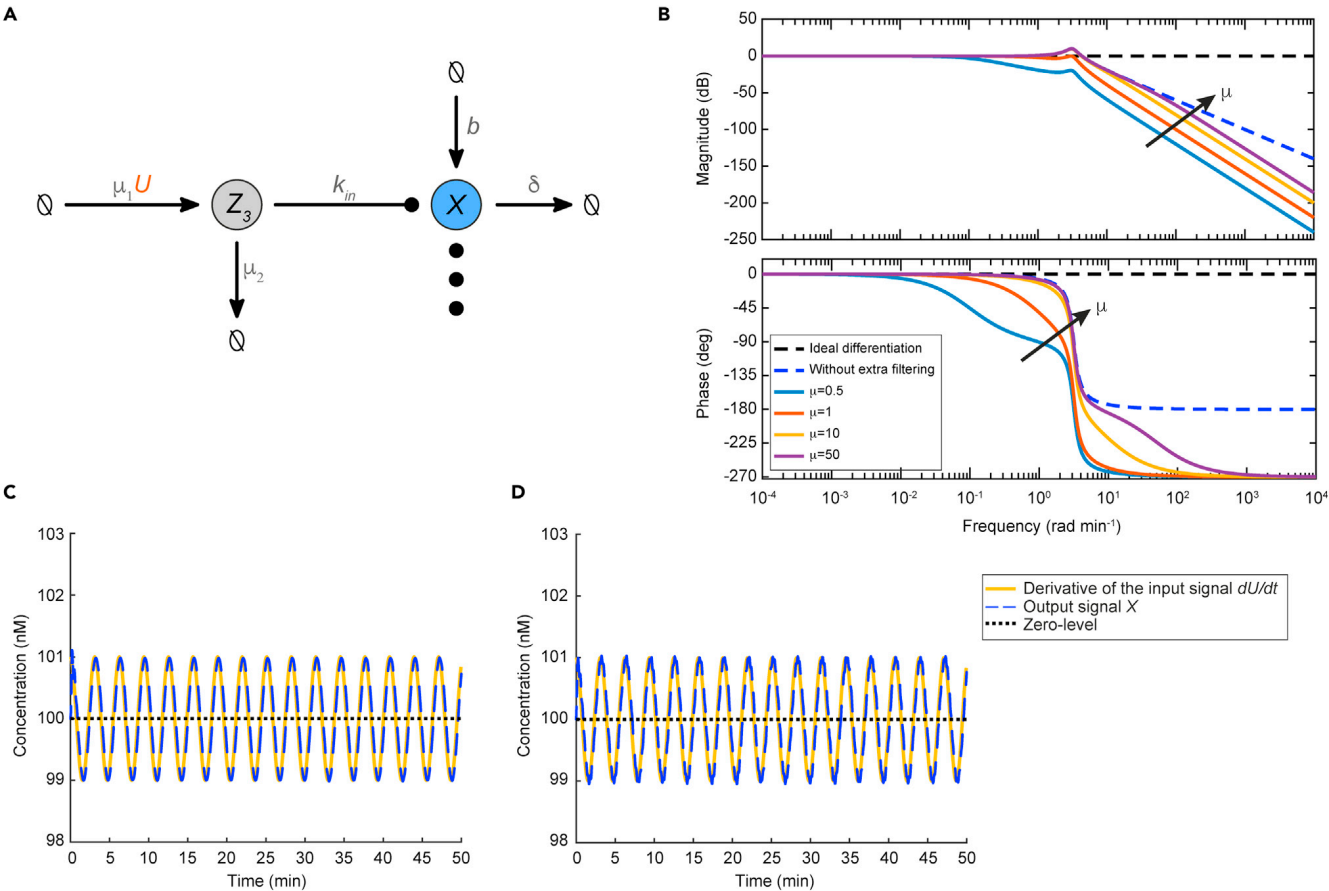
An alternative version of Biomolecular Signal Differentiators with an enhanced capability of input noise filtering (A) Schematic structure of BioSD^*F*^. We adopt the same arrow notation as in Figure 1 while the symbol (⋮) represents the remaining reactions composing any of the three BioSD devices. (B) Bode plot of the performance metric given by Equation (25) with ε=0.1. We consider different values of *μ*, where $\mu =\mu_{1}=\mu_{2}$, that correspond to solid lines of different colors while the increasing direction of *μ* indicated by an arrow. We also depict the bode plot (magnitude and phase) of Equation (14) for the same value of *ε* and the case of ideal differentiation which are represented by blue and black dashed lines, respectively. In addition, for comparison purposes, we focus on a BioSD^*F*^ device based on BioSD-I to re-plot the simulation of **c**Figures 5A and **d**Figure 5B for the same values of the mutual parameters and $\mu_{1}=\mu_{2}=5$ min^−1^. It is apparent that in both (C) and (D) very strong input noise attenuation takes place and the differentiation of the useful signal is thus conducted with significantly high accuracy.

In case there are increased requirements for noise reduction that cannot be easily met via parameter tuning, we present an alternative version of the BioSD networks with higher noise insensitivity, which we call BioSD^*F*^ (Figure 6A). These topologies are described by the same CRNs presented in the section Biological structure, but amended appropriately.

More analytically, recalling the CRNs given by Equations (1), (2), and (3), we see that input signals are applied to BioSD modules through the reaction:

$$\emptyset \overset{k_{in}U}{\to }X$$

In BioSD^*F*^ topologies, the above is replaced by the following set of reactions:

$$\emptyset \overset{\mu_{1}U}{\to }Z_{3},\;Z_{3}\overset{k_{in}}{\to }Z_{3}+X,\;Z_{3}\overset{\mu_{2}}{\to }\emptyset$$

The additional species $Z_{3}$ is produced by the input species and degrades in the traditional manner while it catalyzes the formation of the output species. This structural addition is inspired by the work in (Samoilov et al., 2002; Laurenti et al., 2018), where biomolecular concepts from the area of signal processing were studied. In the following, we briefly present the main features of BioSD^*F*^ modules – a comprehensive analysis of their behavior can be found in STAR Methods An alternative version of biomolecular signal differentiators (Figures S4 and S5).

The input/output relation of BioSD^*F*^ networks in the Laplace domain can be described by the transfer function:

(Equation 24) $${\tilde{\text{Δ}}}_{BSD^{F}}\left(s\right)=\frac{\mu_{1}}{s+\mu_{2}}\cdot \frac{s}{\varepsilon \left(s^{2}+s\right)+1}$$

Similarly to BioSDs, we introduce the (normalized) performance metric:

(Equation 25) $${\tilde{\text{Λ}}}_{F}\left(j\omega \right)=\frac{\mu_{2}}{\mu_{1}}\frac{{\tilde{X}}_{nF}\left(j\omega \right)}{{\tilde{X}}_{nd}\left(j\omega \right)}=\frac{\mu_{2}}{j\omega +\mu_{2}}\frac{1}{\varepsilon \left(-\omega^{2}+j\omega \right)+1}$$

where${\tilde{X}}_{nF}\left(j\omega \right)$ refers to the output of a BioSD^*F*^ network.

Using the magnitude-phase representation of Equation (25) we get:

(Equation 26) $$\left|{\tilde{\text{Λ}}}_{F}\left(j\omega \right)\right|=\frac{1}{\sqrt{1+{\left(\frac{\omega }{\mu_{2}}\right)}^{2}}}\frac{1}{\sqrt{\varepsilon^{2}\omega^{2}+{\left(1-\varepsilon \omega^{2}\right)}^{2}}}$$

and

$$∠{\tilde{\text{Λ}}}_{F}\left(j\omega \right)=\text{arctan}\left(\frac{-\varepsilon \omega }{1-\varepsilon \omega^{2}}\right)+\text{arctan}\left(\frac{-\omega }{\mu_{2}}\right)$$

When ${\tilde{\text{Λ}}}_{F}\left(j\omega \right)$ is close to $1∠0^{\circ }$ signal differentiation of high accuracy is achieved (Figure 6B) and the BioSD^*F*^ output can be approximated by:

(Equation 27) $$X=\frac{\mu_{1}k_{in}}{\mu_{2}k_{1}k_{3}}\dot{U}+\frac{k_{3}}{k_{2}}$$

Compared to the original BioSD topologies (Equation (16)), we now have two additional tuning parameters ($\mu_{1}$, $\mu_{2}$) with respect to the output differentiation gain when it comes to the low-frequency regime. However, the major advantage of this version of differentiators is an enhanced capability of noise filtering. In fact, we can have a greatly extended frequency range across which very strong attenuation of high frequency input noise can be achieved (Figures 6C and 6D). In that case, Equation (26) approaches zero. At the same time, the width of this frequency band depends on $\mu_{2}$ and can be adjusted appropriately. As Equation (24) immediately reveals, the latter advantage stems from the fact that compared to BioSD circuits, BioSDs^F^ are equipped with an additional low-pass filter.

### Biomolecular signal differentiators in natural regulatory networks

As outlined in the introduction, derivative action appears to be an important mechanism in various biological systems. To explore the biological relevance of the proposed BioSDs for cellular adaptations to environmental changes, we identified two naturally occurring and well-investigated regulatory network motifs that resemble the BioSD-II network. Note that these natural topologies are operating in the larger context of complex regulatory networks involving a plethora of signaling factors, some of which remain to be identified. We therefore describe the relevant motifs but do not comprehensively detail all interactions occurring in the biological system.

#### Stationary phase and starvation response - RpoS regulatory network

**Figure 7:**
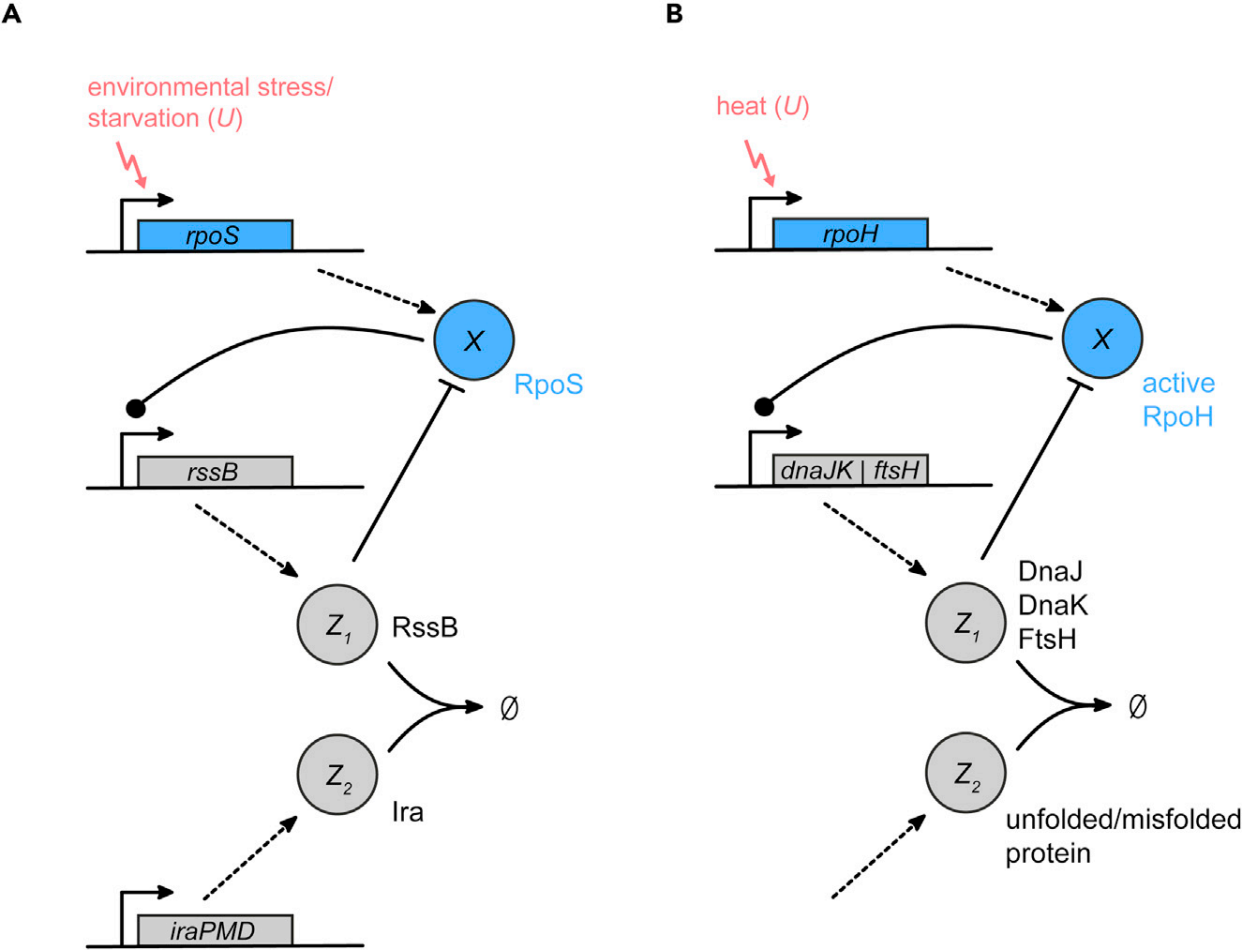
Examples of the Biomolecular Signal Differentiator-II motif in natural systems Simplified schematics of BioSD-II topologies occurring as part of (A) the RpoS-mediated stress response and (B) the RpoH-mediated heat shock response in *Escherichia coli*. Corresponding components of BioSD-II are indicated.

As shown in Figure 7A, we found the BioSD-II motif in the context of adaptation to nutrient starvation and entry into stationary phase, which is mediated by the sigma factor RpoS in *E*. *coli* and related bacteria (reviewed in (Battesti et al., 2011; Hengge-Aronis, 2002)). Stress conditions, such as nutrient depletion or high pH, serve as the input *U*. While RpoS is present at low levels (*b*) in exponentially growing cells, its expression is substantially increased through both transcriptional and post-transcriptional regulation in response to environmental stresses or starvation (Battesti et al., 2011). One of the genes whose expression is dependent on RpoS is *rssB*, which encodes a response regulator. RssB binds to RpoS and mediates its degradation by the ClpXP protease (Pruteanu and Hengge-Aronis, 2002), thus functioning as $Z_{1}$. Nutrient starvation also induces the expression of several anti-adaptor proteins (Ira; inhibitor of RssB activity). These proteins bind to RssB and prevent RpoS degradation (Battesti et al., 2013), which corresponds to the action of $Z_{2}$ in BioSD-II.

#### Heat shock response - RpoH regulatory network

A second example for the BioSD-II motif was identified in the regulatory network of the sigma factor RpoH, which coordinates the heat shock response in *E*. *coli* (Figure 7B) (Straus et al., 1987; Roncarati and Scarlato, 2017). Upon heat shock, cellular RpoH levels rise above their low baseline concentration (*b*), inducing the expression of several chaperones (e.g. DnaKJ and GroELS) and proteases (e.g. FtsH and Lon). DnaK and DnaJ can bind to RpoH and facilitate its degradation by FtsH (Straus, Walter, and Gross, 1989a; Gamer et al., 1992), thereby acting as $Z_{1}$. Unfolded or misfolded proteins will sequester chaperones and proteases (Gamer et al., 1992), thus increasing the stability of RpoH and fulfilling the function of $Z_{2}$. In this network, the amount of *active* RpoH (as opposed to the total amount of RpoH) should be considered as *X*, since it has been found that the activity rather than the concentration of RpoH inside the cell drops during temperature downshifts (Straus, Walter, and Gross, 1989b).

### Guidelines for experimental implementation of biomolecular signal differentiators

**Figure 8:**
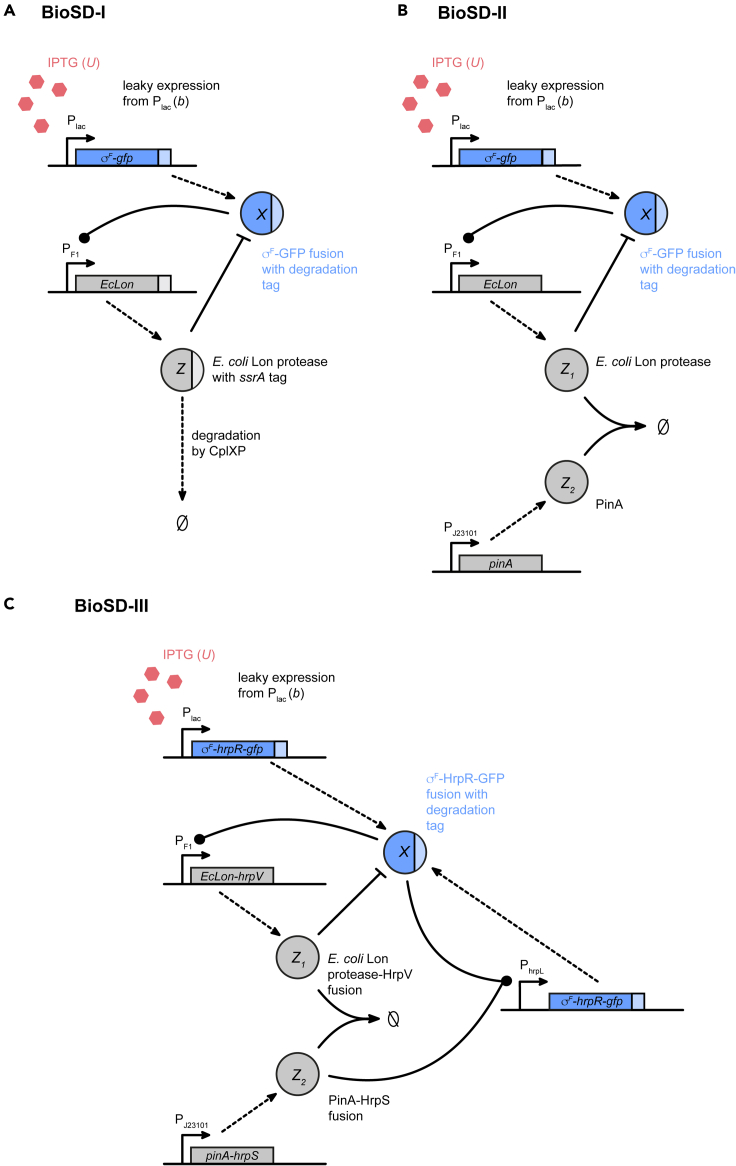
Possible experimental implementations of Biomolecular Signal Differentiators Schematic representation of synthetic designs for ABioSD-I, BBioSD-II and CBioSD-III.

In addition to the natural regulatory networks described in the preceding section, here we outline possible synthetic implementations for all BioSD circuits inside a living cell and, in particular, in *E*. *coli* (Figure 8). Inducible expression of species *X* can be achieved from any well-characterized promoter, such as the IPTG-inducible P_lac_. Leakiness of the lac promoter will ensure nonzero expression levels (*b*) even in the absence of inducer. Alternatively, if higher baseline expression levels are required, *X* could additionally be expressed from a weak constitutive promoter. To minimize undesirable interference with other cellular processes, *X* should be an orthogonal sigma factor, such as $\sigma^{F}$ from *Bacillus subtilis* (Bervoets et al., 2018). A translational fusion of *X* to GFP will allow for easy tracking of the system output. $\sigma^{F}$will then induce expression of a Lon protease (*Z* in BioSD-I, $Z_{1}$ in BioSD-II and III) from its cognate promoter P_F1_. In this case, a Lon^−^ strain of *E*. *coli* would be used to avoid interference of naturally present Lon protease. Addition of a degradation tag to $\sigma^{F}$ will target it for degradation by the Lon protease. To approximate 0th-order degradation of *Z* in BioSD-I, an *ssrA* tag will be fused to the Lon protease as described in (Wong et al., 2007; Ang et al., 2010).

For BioSD-II, we additionally introduce constitutive expression of the protease inhibitor PinA from phage T4 ($Z_{2}$), which has been shown to specifically inhibit the Lon protease in *E*. *coli* with high affinity (Hilliard et al., 1998). A synthetic promoter from the BioBrick collection (Kelly et al., 2009) may be used to achieve the desired expression level of $Z_{2}$. Ideally, an orthogonal Lon protease should be used (e.g. Lon protease from *Mesoplasma florum* (Aoki et al., 2019)) to prevent cross-talk with other cellular proteins. However, since the interaction of PinA with proteases has been characterized only in *E*. *coli* so far, we have suggested use of the *E*. *coli* Lon protease.

Due to the number of required interactions in BioSD-III, it will likely be necessary to introduce auxiliary species for *X*, $Z_{1}$and $Z_{2}$, which we refer to as $X_{aux}$, $Z_{1,aux}$ and $Z_{2,aux}$, respectively. These auxiliary species would ideally have identical behavior to the main species *X*, $Z_{1}$and $Z_{2}$, even though simulations indicate that completely identical behavior is not required (see STAR Methods Analysis of the experimental topology of Biomolecular Signal Differentiator-III and Figure S6). One option is to augment the design for BioSD-II with the Hrp system from *Pseudomonas syringae*, which has previously been implemented in synthetic biology studies (Wang et al., 2014). HrpR ($X_{aux}$) is expressed from P_lac_ together with $\sigma^{F}$, and HrpS ($Z_{2,aux}$) is expressed as a protein fusion with PinA. HrpR and HrpS are both required to induce additional production of $\sigma^{F}$ and HrpR from P_hrpL_. At the same time, HrpV ($Z_{1,aux}$) binds HrpS rendering it inactive. The structural addition required for BioSD^*F*^ can be implemented by, for example, expressing *X* from a T7 promoter and expressing T7 RNA polymerase ($Z_{3}$) from a separate inducible promoter.

## Discussion

In this study, we propose three biomolecular topologies that are able to act as highly accurate signal differentiators inside the cell. These designs provide guidance for building cellular devices capable of computing time derivatives of molecular signals. At the same time, they reveal concepts that are found in natural biological networks implementing differentiation and derivative feedback.

More specifically, we introduce three general biomolecular architectures BioSD-I, II, and III. Their generality lies in the fact that they are represented by CRNs without being restricted by the biological identity of reactants and products and, by extension, the corresponding biological pathway. Important structural components of the BioSDs are a negative feedback loop created by a special process of excitation and inhibition between two species (Iglesias and Shi, 2014), an enzymatic degradation of zero-order kinetics (BioSD-I), an autocatalytic-like reaction (BioSD-III) and an antithetic-like motif based on annihilation (Oishi and Klavins, 2011; Briat et al., 2016) (BioSD-II, BioSD-III). We theoretically analyze their features and show the conditions under which high performance can be guaranteed. Among others, important concepts such as stability, tunability, and accuracy are discussed in detail.

Special emphasis is placed on the expected sensitivity of differentiators to input signals corrupted by high-frequency noise. We demonstrate that this issue can be resolved to a certain extent through suitable parameter tuning. Nevertheless, for cases in which stronger noise attenuation is needed, we present a structural modification that gives rise to three slightly different architectures, namely BioSD^*F*^-I, II and III, with enhanced capabilities. However, the price for this improvement is the addition of an extra biomolecular species, which implies an increase in structural complexity. Moreover, we introduce performance metrics both for BioSDs and BioSDs^F^ based on which the circuit designer can assess the quality of signal differentiation and attenuation. These metrics take into account both the frequency content of the input signal and the reaction rates involved in the circuits, thus facilitating tuning according to the expected performance standards.

The ability to perform time differentiation is of central importance in various biological systems, contributing to stability and fast adaptation to changing conditions (Barkai and Leibler, 1997; Bazellières et al., 2015; Cloutier and Wellstead, 2010). Owing to the generality of the presented topologies, we anticipate that the present study will facilitate the investigation of naturally occurring systems capable of derivative action. In this study, we discuss the regulatory networks of two bacterial sigma factors, RpoS and RpoH, which play a central role in the response and adaptation to stress conditions and heat shock, respectively. Interestingly, these networks share structural characteristics with one of the proposed topologies, BioSD-II.

In addition, the motifs presented here provide building blocks that can be both implemented in stand-alone applications, such as speed biosensors, and also combined with existing biochemical control structures in a modular fashion, e.g., for building biomolecular PID controllers (Chevalier et al., 2019). We describe potential designs for synthetic experimental implementation of all three BioSDs, which can be readily adapted depending on the nature of the system and available biological parts. To realize the antithetic motif in BioSD-II and III, we propose the use of a protease/protease inhibitor pair as an alternative to the previously described systems using sigma and anti-sigma factors (Aoki et al., 2019) or sRNAs and mRNAs (Huang et al., 2018; Kelly et al., 2018). To allow for greater flexibility in choosing the biomolecular species, we introduce a concept of auxiliary species whose usefulness is demonstrated through BioSD-III. Furthermore, to enhance the biological significance of our work in STAR Methods Modeling a more realistic case of Biomolecular Signal Differentiator-II (Figures S7 and S8, Table S1), we investigate the behavior of one of the differentiator modules, namely BioSD-II, under more realistic conditions stemming from our experimental designs.

Stochasticity is an essential characteristic of biomolecular systems which operate in a noisy environment (Del Vecchio and Murray, 2015; Laurenti et al., 2018; Raj and Van Oudenaarden, 2008; Eldar and Elowitz, 2010; Cardelli et al., 2016; Warne et al., 2019). The biomolecular motifs introduced in the current study were analyzed through ODE models (deterministic analysis) which generally approximate well the dynamics of CRNs whose species are present in high copy-numbers. It therefore remains an interesting endeavor to identify the probabilistic effects of the molecular reactions involved that may have a significant impact on the behavior of these motifs when the biomolecular counts are low.

The speed or higher derivatives of the output of a system offers important information about its properties. For an electromechanical system this is not difficult, but it has been a challenging question for biological systems. In this article, we provide an approach to gain access to this information, which will be invaluable for assessing and improving the performance of biological systems. We believe that our BioSD topologies will expand the tools available for understanding and engineering biological systems for robustness and reliability.

### Limitations of the study

As emphasized in the Discussion, the behavior of the topologies presented here is studied via deterministic mathematical analysis and simulations; the effect of inherent stochasticity of living systems stemming from the random nature of molecular reactions on these topologies is left for future work.

## Supporting citations

The following references appear in the supplemental information: Buchler and Louis, 2008; Fekkes et al., 1995; Gur et al., 2012; Schlosshauer and Baker, 2004.

## STAR★Methods

### Key resources table

**Table:** 

REAGENT or RESOURCE	SOURCE	IDENTIFIER
**Software and algorithms**		

MATLAB	Mathworks	www.mathworks.com
Matlab code used for simulations	This study	https://github.com/emgalox/BioS-Differentiators

### Resource availability

#### Lead contact

Further information and requests for resources and reagents should be directed to and will be fulfilled by the lead contact, Antonis Papachristodoulou (antonis@eng.ox.ac.uk).

#### Materials availability

This study did not generate new unique materials.

### Method details

#### Signals under consideration

In this study we consider Fourier-transformable signals (unless otherwise stated) (Lathi, 1998; Oppenheim et al., 1996). The Fourier transform exists for any signal, s(t), satisfying the following conditions, also known as Dirichlet conditions:

• s(t) is absolutely integrable, i.e.:

$$\int_{-\infty }^{+\infty }\left|s\left(t\right)\right|dt<\infty$$

• s(t)has a finite number of maxima and minima within any finite interval.
• s(t)has a finite number of discontinuities within any finite interval. In addition, each of these discontinuities must be finite.

The Dirichlet conditions are sufficient but not necessary for the existence of Fourier transform of a signal. Moreover, it should be noted that the Fourier transform of periodic signals can be computed from their Fourier series representation (assuming it exists) with the help of impulse functions.

The main idea behind Fourier analysis is the decomposition of a signal into a sum of sinusoids, the relative amplitudes and phases of which are determined by the Fourier spectrum of that signal. In the case of a linear, time invariant system, transmission of a signal can be therefore treated as transmission of its constituent sinusoids. Moreover, the frequency-domain description of such a system using its frequency response is an alternative to the time-domain description based on convolution.

Finally, in the current study we focus on physical signals that can be generated in a cellular environment. Such naturally-occurring signals typically satisfy the Dirichlet conditions and, thus, have a Fourier representation - signals that do not satisfy these conditions do not normally arise in practical applications. Further details on the above can be found in (Lathi, 1998; Oppenheim et al., 1996).

#### Equilibria and stability of biomolecular signal differentiators

We assume that all biomolecular circuits in this study are represented by chemical reaction networks (CRNs) whose dynamics are described by the law of mass action unless otherwise stated. For the purposes of deterministic modeling, we consider inputs U(t) that are bounded, non-negative, continuous-time signals of finite duration, the time derivatives of which exist and are also bounded and continuous. This is clearly aligned with the biological nature of U(t) which can correspond, for example, to the concentration of a biomolecular species.

##### Biomolecular signal differentiator-I

Biomolecular Signal Differentiator-I (BioSD-I) is described by the CRN:

(Equation S1) $$\begin{array}{c} \emptyset \overset{k_{in}U}{\to }X,\;\emptyset \overset{b}{\to }X,\;X\overset{k_{2}}{\to }X+Z\\ X+Z\overset{k_{1}}{\to }Z,\;X\overset{\delta }{\to }\emptyset ,\;Z\overset{k_{3}/Z}{\to }\emptyset \end{array}$$

where $k_{in}$, *b*, $k_{2}$, $k_{1}$, $k_{3}$, *δ* $\in R_{+}$. Note that the removal rate of *Z* is constant and equal to $k_{3}$. To achieve this we assume that *Z* participates in an enzyme-catalyzed degradation process which is traditionally described by Michaelis-Menten kinetics. More precisely, the removal rate of *Z* is equal to

(Equation S2) $$k_{3}\frac{Z}{Z+K_{m}}$$

where $K_{m}\;\in R_{+}$ is the Michaelis-Menten constant. When the enzyme that catalyzes the degradation process is saturated by its substrate, we have:

(Equation S3) $$Z\gg K_{m}$$

which entails, in effect, zero-order kinetics since Equation (S2) becomes practically equal to $k_{3}$.

The dynamics of the above CRN (Equation (S1)) are given by the following system of Ordinary Differential Equations (ODEs):

(Equation S4) $$\dot{X}=k_{in}U+b-k_{1}XZ-\delta X$$

(Equation S5) $$\dot{Z}=k_{2}X-k_{3}$$

For any constant input $U^{\text{∗}}$, a steady state ($X^{\text{∗}}$, $Z^{\text{∗}}$) of the system given by Equations (S4) and (S5) exists and is finite. By setting the time derivatives of this system to zero, we can obtain the following unique steady-state:

(Equation S6) $$X^{\text{∗}}=\frac{k_{3}}{k_{2}}$$

(Equation S7) $$Z^{\text{∗}}=\frac{k_{2}\left(k_{in}U^{\text{∗}}+b\right)}{k_{1}k_{3}}-\frac{\delta }{k_{1}}$$

Clearly $X^{\text{∗}}$ is positive while, due to Equation (S3), the same is true for $Z^{\text{∗}}$ (in fact: $Z^{\text{∗}}\gg 0$).

To study the local stability of the above equilibrium, we linearize Equations (S4) and (S5) around ($X^{\text{∗}}$, $Z^{\text{∗}}$) for a constant input $U^{\text{∗}}$ to get:

(Equation S8) $$\left[\begin{array}{l} \dot{X}\\ \dot{Z} \end{array}\right]=\underset{G_{1}}{\underset{︸}{\left[\begin{array}{cc} -\frac{k_{2}\left(k_{in}U^{\text{∗}}+b\right)}{k_{3}} & -\frac{k_{1}k_{3}}{k_{2}}\\ k_{2} & 0 \end{array}\right]}}\left[\begin{array}{l} X\\ Z \end{array}\right]$$

As far as the linear system described by Equation (S8) is concerned, the steady state ($X^{\text{∗}}$, $Z^{\text{∗}}$) is exponentially stable since matrix $G_{1}$ is Hurwitz. To prove this, we find the characteristic polynomial of $G_{1}$ as:

(Equation S9) $$P_{1}\left(s\right)=\text{det}\left(sI-G_{1}\right)=s^{2}+\frac{k_{2}}{k_{3}}\left(k_{in}U^{\text{∗}}+b\right)s+k_{1}k_{3}$$

According to Routh-Hurwitz criterion, the second-order polynomial given by Equation (S9) has both roots in the open left half plane if, and only if, both $\frac{k_{2}\left(k_{in}U^{\text{∗}}+b\right)}{k_{3}}$ and $k_{1}k_{3}$ are positive, which is always true. Consequently, ($X^{\text{∗}}$, $Z^{\text{∗}}$) is a positive locally exponentially stable steady state for the nonlinear system given by Equations (S4) and (S5).

Following the same procedure, we next analyze BioSD-II and BioSD-III.

##### Biomolecular signal differentiator-II

The CRN that corresponds to Biomolecular Signal Differentiator-II (BioSD-II) is:

(Equation S10) $$\begin{array}{c} \emptyset \overset{k_{in}U}{\to }X,\;\emptyset \overset{b}{\to }X,\;X\overset{k_{2}}{\to }X+Z_{1}\\ X+Z_{1}\overset{k_{1}}{\to }Z_{1},\;\emptyset \overset{k_{3}}{\to }Z_{2},\;Z_{1}+Z_{2}\overset{\eta }{\to }\emptyset ,\;X\overset{\delta }{\to }\emptyset \end{array}$$

where $k_{in}$, *b*, $k_{2}$, $k_{1}$, *δ*, *η* $\in R_{+}$.

The dynamics of the above CRN (Equation (S10)) are described by the set of ODEs:

(Equation S11) $$\dot{X}=k_{in}U+b-k_{1}XZ_{1}-\delta X$$

(Equation S12) $${\dot{Z}}_{1}=k_{2}X-\eta Z_{1}Z_{2}$$

(Equation S13) $${\dot{Z}}_{2}=k_{3}-\eta Z_{1}Z_{2}$$

For any constant input $U^{\text{∗}}$, provided that:

(Equation S14) $$k_{2}\left(k_{in}U^{\text{∗}}+b\right)>\delta k_{3},$$

we have a unique positive (finite) steady state:

(Equation S15) $$X^{\text{∗}}=\frac{k_{3}}{k_{2}}$$

(Equation S16) $$Z_{1}^{\text{∗}}=\frac{k_{2}\left(k_{in}U^{\text{∗}}+b\right)}{k_{1}k_{3}}-\frac{\delta }{k_{1}}$$

(Equation S17) $$Z_{2}^{\text{∗}}=\frac{k_{3}}{\eta \left(\frac{k_{2}\left(k_{in}U^{\text{∗}}+b\right)}{k_{1}k_{3}}-\frac{\delta }{k_{1}}\right)}$$

We now linearize Equations (S11), (S12), and (S13) around the fixed point defined by Equations (S15), (S16), and (S17) to obtain:

$$\left[\begin{array}{l} \dot{X}\\ {\dot{Z}}_{1}\\ {\dot{Z}}_{2} \end{array}\right]=\underset{G_{2}}{\underset{︸}{\left[\begin{array}{lll} -\frac{k_{2}\left(k_{in}U^{\text{∗}}+b\right)}{k_{3}} & -\frac{k_{1}k_{3}}{k_{2}} & \;0\\ \;\;\;k_{2} & -\eta Z_{2}^{\text{∗}} & -\eta Z_{1}^{\text{∗}}\\ \;\;\;0 & -\eta Z_{2}^{\text{∗}} & -\eta Z_{1}^{\text{∗}} \end{array}\right]}}\left[\begin{array}{l} X\\ Z_{1}\\ Z_{2} \end{array}\right]$$

The characteristic polynomial of $G_{2}$ is:

(Equation S18) $$P_{2}\left(s\right)=\text{det}\left(sI-G_{2}\right)=s^{3}+\alpha_{2}s^{2}+\alpha_{1}s+\alpha_{0}$$

where

(Equation S19) $$\alpha_{2}=\sigma +\eta \left(Z_{1}^{\text{∗}}+Z_{2}^{\text{∗}}\right)$$

(Equation S20) $$\alpha_{1}=k_{1}k_{3}+\sigma \eta \left(Z_{1}^{\text{∗}}+Z_{2}^{\text{∗}}\right)$$

(Equation S21) $$\alpha_{0}=k_{1}k_{3}\eta Z_{1}^{\text{∗}}$$

and

$$\sigma =\frac{k_{2}\left(k_{in}U^{\text{∗}}+b\right)}{k_{3}}$$

The polynomial given by Equation (S18) has all roots in the open-half plane if and only if $\alpha_{2},\alpha_{0}$ are positive and $\alpha_{2}\alpha_{1}>\alpha_{0}$ (Routh-Hurwitz criterion). Indeed:

$$\begin{array}{l} \left(\sigma +\eta \left(Z_{1}^{\text{∗}}+Z_{2}^{\text{∗}}\right)\right)\left(k_{1}k_{3}+\sigma \eta \left(Z_{1}^{\text{∗}}+Z_{2}^{\text{∗}}\right)\right)>\eta k_{1}k_{3}z_{1}^{\text{∗}}\\ \text{or}\\ \sigma k_{1}k_{3}+\sigma^{2}\eta \left(Z_{1}^{\text{∗}}+Z_{2}^{\text{∗}}\right)+k_{1}k_{3}\eta \left(Z_{1}^{\text{∗}}+Z_{2}^{\text{∗}}\right)+\sigma \eta^{2}{\left(Z_{1}^{\text{∗}}+Z_{2}^{\text{∗}}\right)}^{2}>\eta k_{1}k_{3}z_{1}^{\text{∗}}\\ \text{or}\\ \sigma k_{1}k_{3}+\sigma^{2}\eta \left(Z_{1}^{\text{∗}}+Z_{2}^{\text{∗}}\right)+\sigma \eta^{2}{\left(Z_{1}^{\text{∗}}+Z_{2}^{\text{∗}}\right)}^{2}+\eta k_{1}k_{3}Z_{2}^{\text{∗}}>0 \end{array}$$

which is always true since all the quantities involved are positive. Therefore, ($X^{\text{∗}}$, $Z_{1}^{\text{∗}}$, $Z_{2}^{\text{∗}}$) is a positive locally exponentially stable steady state ($G_{2}$ is Hurwitz) for the nonlinear system described by Equations (S11), (S12), and (S13).

Note that outside the parameter regime defined by Equation (S14) BioSD-II is unable to reach equilibrium. In particular, assuming non-negative initial conditions for Equations (S11), (S12), and (S13) (which is always the case because the variables involved represent biomolecular concentrations) the states of the latter remain always non-negative (as expected from mass action kinetics). Indeed, when X=0, Equation (S11) implies $\dot{X}=k_{in}U+b>0$. Furthermore, when $Z_{1}=0$, Equation (S12) results in $\dot{Z_{1}}=k_{2}X\geq 0$ and, finally, when $Z_{2}=0$, Equation (S13) imposes $\dot{Z_{2}}=k_{3}>0$. However, outside the parameter regime in question, one of the following must hold: $k_{2}\left(k_{in}U^{\text{∗}}+b\right)<\delta k_{3}$or $k_{2}\left(k_{in}U^{\text{∗}}+b\right)=\delta k_{3}$. In the first scenario, it is apparent from Equations (S16) and (S17) that the steady state of $Z_{1}$, $Z_{2}$ becomes negative while in the second case Equation (S17) indicates that $Z_{2}$ tends to infinity - thus, BioSD-II cannot approach a finite steady state.

##### Biomolecular signal differentiator-III

Biomolecular Signal Differentiator-III (BioSD-III) is represented by the CRN:

(Equation S22) $$\begin{array}{l} \emptyset \overset{k_{in}U}{\to }X,\;\emptyset \overset{b}{\to }X,\;X\overset{k_{2}}{\to }X+Z_{1},\;X+Z_{1}\overset{k_{1}}{\to }Z_{1}\\ \emptyset \overset{k_{3}}{\to }Z_{2},\;X+Z_{2}\overset{k_{1}}{\to }X+X+Z_{2},\;Z_{1}+Z_{2}\overset{\eta }{\to }\emptyset ,\;X\overset{\delta }{\to }\emptyset \end{array}$$

where $k_{in}$, *b*, $k_{2}$, $k_{1}$, *δ*, *η*$\in R_{+}$.

The corresponding ODE model describing the dynamics is

(Equation S23) $$\dot{X}=k_{in}U+b-k_{1}XZ_{1}+k_{1}XZ_{2}-\delta X$$

(Equation S24) $${\dot{Z}}_{1}=k_{2}X-\eta Z_{1}Z_{2}$$

(Equation S25) $${\dot{Z}}_{2}=k_{3}-\eta Z_{1}Z_{2}$$

For any constant input $U^{\text{∗}}$, we have a unique positive steady state (providing that it exists and is finite):

(Equation S26) $$X^{\text{∗}}=\frac{k_{3}}{k_{2}}$$

(Equation S27) $$Z_{1}^{\text{∗}}=\frac{1}{2}\left[\frac{k_{2}\left(k_{in}U^{\text{∗}}+b\right)}{k_{1}k_{3}}-\frac{\delta }{k_{1}}\right]+\frac{1}{2}\sqrt{{\left[\frac{k_{2}\left(k_{in}U^{\text{∗}}+b\right)}{k_{1}k_{3}}-\frac{\delta }{k_{1}}\right]}^{2}+4\frac{k_{3}}{n}}$$

(Equation S28) $$Z_{2}^{\text{∗}}=-\frac{1}{2}\left[\frac{k_{2}\left(k_{in}U^{\text{∗}}+b\right)}{k_{1}k_{3}}-\frac{\delta }{k_{1}}\right]+\frac{1}{2}\sqrt{{\left[\frac{k_{2}\left(k_{in}U^{\text{∗}}+b\right)}{k_{1}k_{3}}-\frac{\delta }{k_{1}}\right]}^{2}+4\frac{k_{3}}{n}}$$

Linearizing the system given by Equations (S23), (S24), and (S25) around its steady state (Equations (S26), (S27), and (S28)) yields:

$$\left[\begin{array}{l} \dot{X}\\ {\dot{Z}}_{1}\\ {\dot{Z}}_{2} \end{array}\right]=\underset{G_{3}}{\underset{︸}{\left[\begin{array}{lll} -\frac{k_{2}\left(k_{in}U^{\text{∗}}+b\right)}{k_{3}} & -\frac{k_{1}k_{3}}{k_{2}} & \frac{k_{1}k_{3}}{k_{2}}\\ k_{2} & -\eta Z_{2}^{\text{∗}} & -\eta Z_{1}^{\text{∗}}\\ 0 & -\eta Z_{2}^{\text{∗}} & -\eta Z_{1}^{\text{∗}} \end{array}\right]}}\left[\begin{array}{l} X\\ Z_{1}\\ Z_{2} \end{array}\right]$$

The characteristic polynomial of $G_{3}$ is:

(Equation S29) $$P_{3}\left(s\right)=\text{det}\left(sI-G_{3}\right)=s^{3}+\alpha {\text{′}}_{2}s^{2}+\alpha {\text{′}}_{1}s+\alpha {\text{′}}_{0}$$

where $\alpha {\text{′}}_{2}$, $\alpha {\text{′}}_{1}$ are identical to $\alpha_{2}$ (Equation S19), $\alpha_{1}$ (Equation S20), respectively and:

$$\alpha {\text{′}}_{0}=k_{1}k_{3}\eta \left(Z_{1}^{\text{∗}}+Z_{2}^{\text{∗}}\right)$$

In order to show that $G_{3}$ is Hurwitz we need to verify that $\alpha {\text{′}}_{2}\alpha {\text{′}}_{1}>\alpha {\text{′}}_{0}$ (from Routh-Hurwitz criterion).

This inequality is satisfied because:

$$\begin{array}{l} \left(\sigma +\eta \left(Z_{1}^{\text{∗}}+Z_{2}^{\text{∗}}\right)\right)\left(k_{1}k_{3}+\sigma \eta \left(Z_{1}^{\text{∗}}+Z_{2}^{\text{∗}}\right)\right)>\eta k_{1}k_{3}\left(Z_{1}^{\text{∗}}+Z_{2}^{\text{∗}}\right)\\ \text{or}\\ \sigma k_{1}k_{3}+\sigma^{2}\eta \left(Z_{1}^{\text{∗}}+Z_{2}^{\text{∗}}\right)+k_{1}k_{3}\eta \left(Z_{1}^{\text{∗}}+Z_{2}^{\text{∗}}\right)+\sigma \eta^{2}{\left(Z_{1}^{\text{∗}}+Z_{2}^{\text{∗}}\right)}^{2}>k_{1}k_{3}\eta \left(Z_{1}^{\text{∗}}+Z_{2}^{\text{∗}}\right)\\ \text{or}\\ \sigma k_{1}k_{3}+\sigma^{2}\eta \left(Z_{1}^{\text{∗}}+Z_{2}^{\text{∗}}\right)+\sigma \eta^{2}{\left(Z_{1}^{\text{∗}}+Z_{2}^{\text{∗}}\right)}^{2}>0 \end{array}$$

which is always true as a sum of positive quantities. Hence, ($X^{\text{∗}}$, $Z_{1}^{\text{∗}}$, $Z_{2}^{\text{∗}}$) is a positive locally exponentially stable steady state for the nonlinear system described by Equations (S23), (S24), and (S25).

#### The notion of strong rate of annihilation between *Z*_1_, *Z*_2_ (large *η*) in Biomolecular Signal Differentiator-II

This reaction describes a process where species $Z_{1}$, $Z_{2}$ bind to each other irreversibly to form a product which can be considered as biologically inactive. In other words, this product does not participate in any of the reactions in BioSD-II. Here we demonstrate that the steady state of $Z_{2}$ as well as its deviation from it is practically negligible if the formation rate, *η*, of the product in question is sufficiently high. At the same time, the effect of $Z_{2}$ on the dynamics of BioSD-II can be considered insignificant, too.

By adopting the coordinate transformations: $u=U-U^{\text{∗}}$, $x=X-X^{\text{∗}}$, $z_{1}=Z_{1}-Z_{1}^{\text{∗}}$, $z_{2}=Z_{2}-Z_{2}^{\text{∗}}$ which denote small perturbations around ($U^{\text{∗}}$, $X^{\text{∗}}$, $Z_{1}^{\text{∗}}$, $Z_{2}^{\text{∗}}$), we obtain through linearization of Equations (S11), (S12), and (S13):

(Equation S30) $$\left[\begin{array}{l} \dot{x}\\ {\dot{z}}_{1}\\ {\dot{z}}_{2} \end{array}\right]=\left[\begin{array}{lll} -\frac{k_{2}\left(k_{in}U^{\text{∗}}+b\right)}{k_{3}} & -\frac{k_{1}k_{3}}{k_{2}} & 0\\ k_{2} & -\eta Z_{2}^{\text{∗}} & -\eta Z_{1}^{\text{∗}}\\ 0 & -\eta Z_{2}^{\text{∗}} & -\eta Z_{1}^{\text{∗}} \end{array}\right]\left[\begin{array}{l} x\\ z_{1}\\ z_{2} \end{array}\right]+\left[\begin{array}{l} k_{in}\\ 0\\ 0 \end{array}\right]u$$

We now introduce the non-dimensional variables:

(Equation S31) $$t_{n}=\beta_{1}t$$

(Equation S32) $$x_{n}=\frac{1}{\beta_{2}}\bar{x}$$

(Equation S33) $$z_{1n}=\frac{\beta_{1}}{\beta_{2}k_{2}}z_{1}$$

(Equation S34) $$z_{2n}=\frac{\beta_{1}}{\beta_{2}k_{2}}z_{2}$$

(Equation S35) $$u_{n}=\frac{k_{in}}{\beta_{1}\beta_{2}}u$$

where

(Equation S36) $$\beta_{1}=\frac{k_{3}}{\left(\frac{k_{2}\left(k_{in}U^{\text{∗}}+b\right)}{k_{1}k_{3}}-\frac{\delta }{k_{1}}\right)}$$

and $\beta_{2}$ is an arbitrary scaling parameter that carries the same units as $x_{n}$. In addition, we introduce the non-dimensional parameters:

(Equation S37) $$\lambda_{1}=\frac{\beta_{1}^{2}}{\eta k_{3}}$$

(Equation S38) $$\lambda_{2}=\frac{k_{2}\left(k_{in}U^{\text{∗}}+b\right)}{\beta_{1}k_{3}}$$

(Equation S39) $$\lambda_{3}=\frac{k_{1}k_{3}}{\beta_{1}k_{2}}$$

By substituting (Equation S31), (Equation S32), (Equation S33), (Equation S34), (Equation S35), (Equation S36), (Equation S37), (Equation S38), and (S39) into the model given by Equation (S30), we obtain:

$${\dot{x}}_{n}=u_{n}-\lambda_{2}x_{n}-\lambda_{3}z_{1n}$$

$${\dot{z}}_{1n}=k_{2}x_{n}-z_{1n}-\frac{1}{\lambda_{1}}z_{2n}$$

$${\dot{z}}_{2n}=-z_{1n}-\frac{1}{\lambda_{1}}z_{2n}$$

or

$${\dot{x}}_{n}=u_{n}-\lambda_{2}x_{n}-\lambda_{3}z_{1n}$$

$$\lambda_{1}{\dot{z}}_{1n}=\lambda_{1}x_{n}-\lambda_{1}z_{1n}-z_{2n}$$

$$\lambda_{1}{\dot{z}}_{2n}=-\lambda_{1}z_{1n}-z_{2n}$$

We now introduce the linear transformation $g_{n}=z_{1n}-z_{2n}$ resulting in the following mathematically equivalent system:

(Equation S40) $${\dot{x}}_{n}=u_{n}-\lambda_{2}x_{n}-\lambda_{3}g_{n}-\lambda_{3}z_{2n}$$

(Equation S41) $${\dot{g}}_{n}=x_{n}$$

(Equation S42) $$\lambda_{1}{\dot{z}}_{2n}=-\lambda_{1}g_{n}-\left(1+\lambda_{1}\right)z_{2n}$$

According to Equation (S37), $\lambda_{1}\to 0$as η→∞. This means that we can make $\lambda_{1}$ negligible by choosing a large value for *η*:

(Equation S43) $$\eta \gg \frac{\beta_{1}^{2}}{k_{3}}$$

We now regard $\lambda_{1}$ as a singular perturbation parameter and use Theorem 11.1 in (H. K. Khalil, 2002). From (Equation S40), (Equation S41), and (S42) we obtain the following reduced model for $\lambda_{1}=0$:

(Equation S44) $${\dot{x}}_{n}=u_{n}-\lambda_{2}x_{n}-\lambda_{3}g_{n}$$

(Equation S45) $${\dot{g}}_{n}=x_{n}$$

since $z_{2n}=0$.

For a finite time interval $\left[0,t_{f}\right]$ of interest, Equations (S44) and (S45) produce a unique solution ${\bar{x}}_{n}\left(t\right),{\bar{g}}_{n}\left(t\right)$ taking into account the initial conditions of the system. In addition, the origin is an exponentially stable equilibrium point of the boundary layer model:

$$\frac{dz_{2n}}{d\tau }=-z_{2n}$$

where $\tau =t_{n}/\lambda_{1}$.

Thus, according to Tikhonov's theorem (Theorem 11.1 in (Khalil, 2002)), there exist a positive constant $\lambda_{1}^{\text{∗}}$ such that for $0<\lambda_{1}<\lambda_{1}^{\text{∗}}$ the singular perturbation problem of (Equation S40), (Equation S41), and (S42) has a unique solution $x_{n}\left(t,\lambda_{1}\right)$, $g_{n}\left(t,\lambda_{1}\right)$, $z_{2n}\left(t,\lambda_{1}\right)$ on $\left[0,t_{f}\right]$ and

$$x_{n}\left(t,\lambda_{1}\right)-{\bar{x}}_{n}\left(t\right)=O\left(\lambda_{1}\right)$$

$$g_{n}\left(t,\lambda_{1}\right)-{\bar{g}}_{n}\left(t\right)=O\left(\lambda_{1}\right)$$

Moreover, given any $t_{b}>0$, there is $\lambda_{1}^{\text{∗∗}}$ such that

$$z_{2n}\left(t,\lambda_{1}\right)=O\left(\lambda_{1}\right)$$

whenever $\lambda_{1}<\lambda_{1}^{\text{∗∗}}$.

Finally, combining (Equation S16), (Equation S17), (Equation S36), and (S37) results in:

$$\lambda_{1}=\frac{Z_{2}^{\text{∗}}}{Z_{1}^{\text{∗}}}$$

Assuming that $Z_{1}^{\text{∗}}$ corresponds to some finite (nonzero) concentration, $Z_{2}^{\text{∗}}\to 0$ as $\lambda_{1}\to 0$.

#### Behavior analysis of biomolecular signal differentiators

Here we prove that, near their equilibria, BioSD networks are capable of signal differentiation.

We begin with BioSD-I whose dynamics close to its steady state are derived via linearization of Equations (S4) and (S5) as:

(Equation S46) $$\left[\begin{array}{l} \dot{x}\\ \dot{z} \end{array}\right]=\left[\begin{array}{ll} -\frac{k_{2}\left(k_{in}U^{\text{∗}}+b\right)}{k_{3}} & -\frac{k_{1}k_{3}}{k_{2}}\\ k_{2} & 0 \end{array}\right]\left[\begin{array}{l} x\\ z \end{array}\right]+\left[\begin{array}{l} k_{in}\\ 0 \end{array}\right]u$$

assuming the coordinate transformations: $u=U-U^{\text{∗}}$, $x=X-X^{\text{∗}}$, $z=Z-Z^{\text{∗}}$ which represent small perturbations around ($U^{\text{∗}}$, $X^{\text{∗}}$, $Z^{\text{∗}}$). Note that *u* represents $U^{TV}$ of the main text. We next consider the non-dimensional variables:

(Equation S47) $$t_{n}=c_{1}t$$

(Equation S48) $$x_{n}=\frac{1}{c_{3}}x$$

(Equation S49) $$z_{n}=\frac{c_{1}}{k_{2}c_{3}}z$$

(Equation S50) $$u_{n}=\frac{c_{1}k_{in}}{k_{2}c_{2}c_{3}}u$$

where

(Equation S51) $$c_{1}=\frac{k_{2}\left(k_{in}U^{\text{∗}}+b\right)}{k_{3}}$$

(Equation S52) $$c_{2}=\frac{k_{1}k_{3}}{k_{2}}$$

and$c_{3}$ is an arbitrary scaling parameter that carries the same units as $x_{n}$. We also introduce the non-dimensional parameter:

(Equation S53) $$\varepsilon =\frac{c_{1}^{2}}{k_{2}c_{2}}$$

Substituting (Equation S47), (Equation S48), (Equation S49), (Equation S50), (Equation S51), (Equation S52) and (S53) into the system (S46) results in:

$${\dot{x}}_{n}=-x_{n}-\frac{1}{\varepsilon }z_{n}+\frac{1}{\varepsilon }u_{n}$$

$${\dot{z}}_{n}=x_{n}$$

or

(Equation S54) $$\varepsilon {\dot{x}}_{n}=-\varepsilon x_{n}-z_{n}+u_{n}$$

(Equation S55) $${\dot{z}}_{n}=x_{n}$$

The system described by Equations (S54), (S55) is mathematically equivalent to the following second - order differential equation:

(Equation S56) $$\varepsilon {\ddot{x}}_{n}+\varepsilon {\dot{x}}_{n}+x_{n}={\dot{u}}_{n}$$

We see immediately that if $\varepsilon \left({\ddot{x}}_{n}+{\dot{x}}_{n}\right)=0$ then $x_{n}={\dot{u}}_{n}$ which gives through (Equation S47), (Equation S48), (Equation S50) and (S52):

(Equation S57) $$x=\frac{k_{in}}{k_{1}k_{3}}\dot{u}$$

By recalling Equation (S6) and our initial coordinate transformations, this relationship can be rewritten as:

(Equation S58) $$X=\frac{k_{in}}{k_{1}k_{3}}\dot{U}+\frac{k_{3}}{k_{2}}$$

Having this in mind and taking into account that *ε* is positive as a combination of positive parameters (Equation (S53)) we calculate the general solution of ${\ddot{x}}_{n}+{\dot{x}}_{n}=0$ as:

(Equation S59) $$x_{n}=\theta_{1}e^{-t_{n}}+\theta_{2}$$

where$\theta_{1},\theta_{2}$ are arbitrary constants. Subsequently, from (Equation S47), (Equation S48), (Equation S57) and (S59) we get:

(Equation S60) $$u=f_{1}e^{-c_{1}t}+f_{2}t+f_{3}$$

where $f_{1}$, $f_{2}$, $f_{3}$ are arbitrary constants.

To study the behavior of BioSD-I in the more general case where the input signal does not satisfy Equation (S60) we consider the following transfer function describing the system defined by Equations (S54) and (S54) in the Laplace domain:

(Equation S61) $${\tilde{\text{Δ}}}_{BSD}\left(s\right)=\frac{{\tilde{X}}_{n}\left(s\right)}{{\tilde{U}}_{n}\left(s\right)}=\frac{s}{\varepsilon \left(s^{2}+s\right)+1}$$

where${\tilde{X}}_{n}\left(s\right)$ and ${\tilde{U}}_{n}\left(s\right)$ are the Laplace transform of the output $x_{n}$ and input $u_{n}$, respectively and *s* is the complex frequency. As can be seen, BioSD-I can compute the derivative of the input signal filtered by a second - order low - pass filter.

As pointed out in Signals under consideration, Fourier transform is a powerful tool that allows the decomposition of a signal into its constituent sinusoids. Thus, focusing on the frequency response of the system, we set s=jω (where $j=\sqrt{-1}$) in Equation (S61) to get:

(Equation S62) $${\tilde{\text{Δ}}}_{BSD}\left(j\omega \right)=\frac{j\omega }{\varepsilon \left(-\omega^{2}+j\omega \right)+1}$$

which can be equivalently represented by:

(Equation S63) $$\left|{\tilde{\text{Δ}}}_{BSD}\left(j\omega \right)\right|=\frac{\omega }{\sqrt{\varepsilon^{2}\omega^{2}+{\left(1-\varepsilon \omega^{2}\right)}^{2}}}$$

and

(Equation S64) $$∠{\tilde{\text{Δ}}}_{BSD}\left(j\omega \right)=\text{arctan}\left(\frac{1}{\varepsilon \omega }-\omega \right)$$

As shown in Figure S3, for a given *ε*, there is a low-frequency range over which BioSD-I functions as a pure signal differentiator and, by extension Equation (S58) holds (the filtering action is practically zero), and a high-frequency one over which it works as a signal attenuator instead. At the same time, there is a narrow frequency band in between where the aforementioned operations may not be carried out with the expected accuracy. The behavior of BioSD-I therefore depends on the value of *ε* as well as on ”how fast” an input signal varies over time.

Following the same procedure, we study the local dynamics of BioSD-III by linearizing Equations (S23), (S24), and (S25):

$$\left[\begin{array}{l} \dot{x}\\ {\dot{z}}_{1}\\ {\dot{z}}_{2} \end{array}\right]=\left[\begin{array}{lll} -\frac{k_{2}\left(k_{in}U^{\text{∗}}+b\right)}{k_{3}} & -\frac{k_{1}k_{3}}{k_{2}} & \frac{k_{1}k_{3}}{k_{2}}\\ k_{2} & -\eta Z_{2}^{\text{∗}} & -\eta Z_{1}^{\text{∗}}\\ 0 & -\eta Z_{2}^{\text{∗}} & -\eta Z_{1}^{\text{∗}} \end{array}\right]\left[\begin{array}{l} x\\ z_{1}\\ z_{2} \end{array}\right]+\left[\begin{array}{l} k_{in}\\ 0\\ 0 \end{array}\right]u$$

where the variables $u=U-U^{\text{∗}}$, $x=X-X^{\text{∗}}$, $z_{1}=Z_{1}-Z_{1}^{\text{∗}}$, $z_{2}=Z_{2}-Z_{2}^{\text{∗}}$ refer to small perturbations around the equilibrium ($U^{\text{∗}}$, $X^{\text{∗}}$, $Z_{1}^{\text{∗}}$, $Z_{2}^{\text{∗}}$). Introducing the linear transformation $g=z_{1}-z_{2}$ results in the following mathematically equivalent system:

(Equation S65) $$\left[\begin{array}{l} \dot{x}\\ \dot{g}\\ {\dot{z}}_{2} \end{array}\right]=\left[\begin{array}{lll} -\frac{k_{2}\left(k_{in}U^{\text{∗}}+b\right)}{k_{3}} & -\frac{k_{1}k_{3}}{k_{2}} & 0\\ k_{2} & 0 & 0\\ 0 & -\eta Z_{2}^{\text{∗}} & -\eta \left(Z_{1}^{\text{∗}}+Z_{2}^{\text{∗}}\right) \end{array}\right]\left[\begin{array}{l} x\\ g\\ z_{2} \end{array}\right]+\left[\begin{array}{l} k_{in}\\ 0\\ 0 \end{array}\right]u$$

We notice that the dynamics of *x* and *g* of the system given by Equation (S65) are identical to that of *x* and *z* of the system given by Equation (S46), respectively. Hence, the output, *x*, of BioSD-III behaves in the exact same way as the one of previously analyzed BioSD-I.

Subsequently, we recall Equation (S30) describing the dynamics of BioSD-II near its equilibrium. It is evident that using the linear transformation $g=z_{1}-z_{2}$ again and assuming a sufficiently large *η* (Equation (S43) holds), the dynamics of *x* and *g* in BioSD-II are described by Equation (S46), namely the dynamics of BioSD-I (see The notion of strong rate of annihilation between Z1, Z2 (large η) in Biomolecular Signal Differentiator-II). By extension, the output behavior of these two circuits is identical.

#### An alternative version of biomolecular signal differentiators

Here we analyze a slightly different version of the previously studied BioSD networks which we call Biomolecular Signal Differentiators^*F*^ (BioSDs^*F*^) that include an additional biomolecular species, $Z_{3}$. In particular, we describe the following three biomolecular topologies:

• **BioSD**^*F*^**-I**

We have the CRN:

$$\begin{array}{c} \emptyset \overset{\mu_{1}U}{\to }Z_{3},\;Z_{3}\overset{k_{in}}{\to }Z_{3}+X,\;Z_{3}\overset{\mu_{2}}{\to }\emptyset ,\;\emptyset \overset{b}{\to }X\\ X\overset{k_{2}}{\to }X+Z,\;X+Z\overset{k_{1}}{\to }Z,\;X\overset{\delta }{\to }\emptyset ,\;Z\overset{k_{3}/Z}{\to }\emptyset \end{array}$$

where $\mu_{1}$, $\mu_{2}$, $k_{in}$, *b*, $k_{2}$, $k_{1}$, *δ*, $k_{3}$ $\in R_{+}$. The 0th-order removal of *Z* is the result of enzymatic degradation following saturated Michaelis - Menten kinetics (see Equilibria and stability of biomolecular signal differentiators: biomolecular signal differentiator-I).

The corresponding ODE model is:

$${\dot{Z}}_{3}=\mu_{1}U-\mu_{2}Z_{3}$$

$$\dot{X}=k_{in}Z_{3}+b-k_{1}XZ-\delta X$$

$$\dot{Z}=k_{2}X-k_{3}$$

• **BioSD**^*F*^**-II**

We have the CRN:

$$\begin{array}{l} \emptyset \overset{\mu_{1}U}{\to }Z_{3},\;Z_{3}\overset{k_{in}}{\to }Z_{3}+X,\;Z_{3}\overset{\mu_{2}}{\to }\emptyset \\ \emptyset \overset{b}{\to }X,\;X\overset{k_{2}}{\to }X+Z_{1},\;X+Z_{1}\overset{k_{1}}{\to }Z_{1}\\ \emptyset \overset{k_{3}}{\to }Z_{2},\;Z_{1}+Z_{2}\overset{\eta }{\to }\emptyset ,\;X\overset{\delta }{\to }\emptyset \end{array}$$

where $\mu_{1}$, $\mu_{2}$, $k_{in}$, *b*, $k_{2}$, $k_{1}$, *δ*, *η* $\in R_{+}$. We assume that the parameter rate *η* is sufficiently large (see The notion of strong rate of annihilation between Z1, Z2 (large η) in Biomolecular Signal Differentiator-II).

The corresponding ODE model is:

$${\dot{Z}}_{3}=\mu_{1}U-\mu_{2}Z_{3}$$

$$\dot{X}=k_{in}Z_{3}+b-k_{1}XZ_{1}-\delta X$$

$${\dot{Z}}_{1}=k_{2}X-\eta Z_{1}Z_{2}$$

$${\dot{Z}}_{2}=k_{3}-\eta Z_{1}Z_{2}$$

• **BioSD**^*F*^**-III**

We have the CRN:

$$\begin{array}{c} \emptyset \overset{\mu_{1}U}{\to }Z_{3},\;Z_{3}\overset{k_{in}}{\to }Z_{3}+X,\;Z_{3}\overset{\mu_{2}}{\to }\emptyset \\ \emptyset \overset{b}{\to }X,\;X\overset{k_{2}}{\to }X+Z_{1},\;X+Z_{1}\overset{k_{1}}{\to }Z_{1},\;\emptyset \overset{k_{3}}{\to }Z_{2}\\ X+Z_{2}\overset{k_{1}}{\to }X+X+Z_{2},\;Z_{1}+Z_{2}\overset{\eta }{\to }\emptyset ,\;X\overset{\delta }{\to }\emptyset \end{array}$$

where $\mu_{1}$, $\mu_{2}$, $k_{in}$, *b*, $k_{2}$, $k_{1}$, *δ*, *η* $\in R_{+}$.

The corresponding ODE model is:

$${\dot{Z}}_{3}=\mu_{1}U-\mu_{2}Z_{3}$$

$$\dot{X}=k_{in}Z_{3}+b-k_{1}XZ_{1}+k_{1}XZ_{2}-\delta X$$

$${\dot{Z}}_{1}=k_{2}X-\eta Z_{1}Z_{2}$$

$${\dot{Z}}_{2}=k_{3}-\eta Z_{1}Z_{2}$$

Each of the above circuits can be seen as the interconnection of two subsystems. More specifically, we have the linear, asymptotically stable, subsystem (the first equation in each of above ODE models):

(Equation S66) $${\dot{Z}}_{3}=\mu_{1}U-\mu_{2}Z_{3}$$

which receives the signal *U* we want to differentiate as input and produces an output $Z_{3}$. This is, in turn, applied as input to a second subsystem whose output is *X*. While the first subsystem is the same in all BioSD^*F*^ topologies, the second one differs. In fact, the latter is identical to BioSD-I, BioSD-II, BioSD-III (see previous sections) for BioSD^*F*^-I, BioSD^*F*^-II, BioSD^*F*^-III, respectively, with the only difference lying in the input, which is now $Z_{3}$ (instead of *U* as before).

For a constant input $U^{\text{∗}}$ the first subsystem defined by Equation (S66) has a unique positive steady state (assuming it exists and is finite):

(Equation S67) $$Z_{3}=\frac{\mu_{1}U^{\text{∗}}}{\mu_{2}}$$

Since Equation (S66) is linear and ($\mu_{2}$) is always positive then Equation (S67) is a globally exponentially stable equilibrium point.

We now concentrate on the local behavior of BioSD^*F*^ modules and, consequently, we consider the coordinate transformations: $u=U-U^{\text{∗}}$, $x=X-X^{\text{∗}}$, $z=Z-Z^{\text{∗}}$, $z_{1}=Z_{1}-Z_{1}^{\text{∗}}$, $z_{2}=Z_{2}-Z_{2}^{\text{∗}}$, $z_{3}=Z_{3}-Z_{3}^{\text{∗}}$ denoting small perturbations around the corresponding equilibria of BioSD^*F*^ networks - ($U^{\text{∗}}$, $X^{\text{∗}}$, $Z^{\text{∗}}$, $Z_{3}^{\text{∗}}$ ) for BioSD^*F*^-I and ($U^{\text{∗}}$, $X^{\text{∗}}$, $Z_{1}^{\text{∗}}$, $Z_{2}^{\text{∗}}$, $Z_{3}^{\text{∗}}$ ) for BioSD^*F*^-II, BioSD^*F*^-III (the steady states of the last two networks do not necessarily coincide).

First, we study Equation (S66) separately. In the Laplace domain, we have:

(Equation S68) $${\tilde{\text{Δ}}}_{LPF}\left(s\right)=\frac{{\tilde{Z}}_{3}\left(s\right)}{\tilde{U}\left(s\right)}=\frac{\mu_{1}}{s+\mu_{2}}$$

where ${\tilde{Z}}_{3}\left(s\right)$, $\tilde{U}\left(s\right)$ are the Laplace transform of $z_{3}$, *u*, respectively. Focusing on the frequency response, we get:

(Equation S69) $${\tilde{\text{Δ}}}_{LPF}\left(j\omega \right)=\frac{\mu_{1}}{\mu_{2}}\frac{1}{j\frac{\omega }{\mu_{2}}+1}$$

This is a transfer function of a first-order low-pass filter which is capable of preserving low-frequency signals and rejecting high-frequency signals. Indeed, the magnitude and the phase of the system in question are given by:

$$\left|{\tilde{\text{Δ}}}_{LPF}\left(j\omega \right)\right|=\frac{\mu_{1}}{\mu_{2}}\frac{1}{\sqrt{1+{\left(\frac{\omega }{\mu_{2}}\right)}^{2}}}$$

and

$$∠{\tilde{\text{Δ}}}_{LPF}\left(j\omega \right)=-\text{arctan}\frac{\omega }{\mu_{2}},$$

respectively.

We can easily see that in practice, when $\omega \ll \mu_{2}$, there is a constant input/output gain $\left(\frac{\mu_{1}}{\mu_{2}}\right)$ and no phase lag. On the other hand, for $\omega^{2}\gg \mu_{2}^{2}$ strong attenuation takes place. The general behavior of the filter can be easily understood through the Bode diagram in Figure S4.

We now consider a BioSD^*F*^ design which can be described by the transfer function of the series connection of the previously studied filter and a BioSD design (as already outlined in Behavior analysis of biomolecular signal differentiators, all three BioSD circuits are described by the same transfer function), i.e.:

$${\tilde{\text{Δ}}}_{BSD^{F}}\left(s\right)=\frac{{\tilde{X}}_{n}\left(s\right)}{{\tilde{U}}_{n}\left(s\right)}={\tilde{\text{Δ}}}_{LPF}\left(s\right){\tilde{\text{Δ}}}_{BSD}\left(s\right)$$

or

(Equation S70) $${\tilde{\text{Δ}}}_{BSD^{F}}\left(s\right)=\frac{\mu_{1}}{s+\mu_{2}}\cdot \frac{s}{\varepsilon \left(s^{2}+s\right)+1}$$

where ${\tilde{\text{Δ}}}_{LPF}\left(s\right)=\frac{{\tilde{Z}}_{3}\left(s\right)}{{\tilde{U}}_{n}\left(s\right)}$, ${\tilde{\text{Δ}}}_{BSD}\left(s\right)=\frac{{\tilde{X}}_{n}\left(s\right)}{{\tilde{Z}}_{3n}\left(s\right)}$ with ${\tilde{Z}}_{3n}\left(s\right)=p{\tilde{Z}}_{3}\left(s\right)$ and $p=\frac{u_{n}}{u}$ (see Behavior analysis of biomolecular signal differentiators).

Shifting our focus on the frequency response we have:

(Equation S71) $${\tilde{\text{Δ}}}_{BSD^{F}}\left(j\omega \right)={\tilde{\text{Δ}}}_{LPF}\left(j\omega \right){\tilde{\text{Δ}}}_{BSD}\left(j\omega \right)$$

for which:

$$\left|{\tilde{\text{Δ}}}_{BSD^{F}}\left(j\omega \right)\right|=\left|{\tilde{\text{Δ}}}_{LPF}\left(j\omega \right)\right|\left|{\tilde{\text{Δ}}}_{BSD}\left(j\omega \right)\right|$$

and

$$∠{\tilde{\text{Δ}}}_{BSD^{F}}\left(\omega \right)=∠{\tilde{\text{Δ}}}_{LPF}\left(j\omega \right)+∠{\tilde{\text{Δ}}}_{BSD}\left(j\omega \right)$$

Consequently, for a given *ε*, BioSD^*F*^ circuits are characterized by an enhanced capability of high-frequency signal attenuation compared to BioSD ones. In fact, as demonstrated in Figure S5, we can extend the frequency band where strong signal attenuation is carried out by appropriately tuning the filter module. In other words, we can adjust the bandwidth of the extra filter as desired through the parameter rate $\mu_{2}$. The price we pay for this significant improvement is the increase in structural complexity due to the addition of the species $Z_{3}$ via which the additional filtering is accomplished. Finally, in the low-frequency regime, where only signal differentiation takes place (the filtering action is practically zero), the BioSD^*F*^ output can be approximated in the time domain as (recall Behavior Analysis of Biomolecular Signal Differentiators):

$$X=\frac{\mu_{1}k_{in}}{\mu_{2}k_{1}k_{3}}\dot{U}+\frac{k_{3}}{k_{2}}$$

#### Analysis of the experimental topology of Biomolecular Signal Differentiator-III

Here we further analyze the proposed synthetic design of BioSD-III, the behavior of which may be more complicated due to the use of three auxiliary species (see Guidelines for experimental implementation of biomolecular signal differentiators).

The biomolecular topology shown in Figure 8C can be described by the following set of ODEs:

(Equation S72) $$\dot{X}=k_{in}U+b-k_{1}XZ_{1}+k_{1a}X_{aux}Z_{2,aux}-\delta X$$

(Equation S73) $${\dot{X}}_{aux}=k_{in}U+b-k_{1b}X_{aux}Z_{1}+k_{1a}X_{aux}Z_{2,aux}-\delta_{a}X_{aux}$$

(Equation S74) $${\dot{Z}}_{1}=k_{2}X-\eta Z_{1}Z_{2}$$

(Equation S75) $${\dot{Z}}_{1,aux}=k_{2}X-\eta_{a}Z_{1,aux}Z_{2,aux}$$

(Equation S76) $${\dot{Z}}_{2}=k_{3}-\eta Z_{1}Z_{2}$$

(Equation S77) $${\dot{Z}}_{2,aux}=k_{3}-\eta_{a}Z_{1,aux}Z_{2,aux}$$

where $k_{in}$, *b*, $k_{2}$, $k_{1}$, $k_{1a}$, $k_{1b}$, *δ*, $\delta_{a}$, *η*, $\eta_{a}$ $\in R_{+}$.

In order for the behavior of *X* (measured output species) in the system described by (Equation S72), (Equation S73), (Equation S74), (Equation S75), (Equation S76), (Equation S77) to perfectly match the one of *X* in the model given by (Equation S23), (Equation S24), (Equation S25), we need: $k_{1}=$$k_{1a}=$$k_{1b}$, δ=$\delta_{a}$ and η=$\eta_{a}$. Nevertheless, non-satisfaction of the aforementioned conditions does not necessarily entail considerable loss of accuracy regarding signal differentiation (Figure S6).

#### Modeling a more realistic case of Biomolecular Signal Differentiator-II

Here we study the behavior of Biomolecular Signal Differentiator-II under more realistic conditions resulting from the corresponding experimental design discussed in Guidelines for experimental implementation of biomolecular signal differentiators.

First, we consider the ODE model:

(Equation S78) $$\dot{X}=k_{in}U+b-k_{1}XZ_{1}-\delta X$$

(Equation S79) $${\dot{Z}}_{1}=V_{max}\frac{X}{X+K_{m}}-\eta Z_{1}Z_{2}$$

(Equation S80) $${\dot{Z}}_{2}=k_{3}-\eta Z_{1}Z_{2}$$

For a constant input $U^{\text{∗}}$, provided that:

$$\left(k_{in}U^{\text{∗}}+b\right)>\delta X^{\text{∗}},$$

we have a unique positive (finite) steady state:

(Equation S81) $$X^{\text{∗}}=\frac{k_{3}K_{m}}{V_{max}-k_{3}}$$

(Equation S82) $$Z_{1}^{\text{∗}}=\frac{\left(k_{in}U^{\text{∗}}+b\right)}{k_{1}X^{\text{∗}}}-\frac{\delta }{k_{1}}$$

(Equation S83) $$Z_{2}^{\text{∗}}=\frac{k_{3}}{\eta Z_{1}^{\text{∗}}}$$

Compared to the original model of BioSD-II (Equations (S11), (S12), and (S13)), we now use a Michaelis-Menten function to describe the activation of species $Z_{1}$ by species *X* (Equation S79) through gene expression (Aoki et al., 2019). It is evident that, assuming small perturbations around ($U^{\text{∗}}$, $X^{\text{∗}}$, $Z_{1}^{\text{∗}}$, $Z_{2}^{\text{∗}}$), linearization of Equations (S78), (S79), and (S80) yields a system of the same form as Equation (S30). Consequently, we can follow a similar analysis to study its local behavior as the one used for the original model (see The notion of strong rate of annihilation between Z1, Z2 (large η) in biomolecular signal differentiator-II and Behavior analysis of biomolecular signal differentiators). Nevertheless, it should be emphasized that when no saturation occurs and the slope of the Michaelis-Menten function is approximately linear, the corresponding production rate can be effectively considered proportional to the concentration of the regulator species (ibid.). In that case, the results of our original analysis can be used directly.

Implementation of BioSD-II in living cells implies the existence of an additional degradation mechanism due to cell growth affecting all the biomolecules involved, known as dilution (Aoki et al., 2019; Qian and Del Vecchio, 2018). This can lead to a “leaky” integration process realized by species $Z_{1}$, $Z_{2}$ and, by extension, it can affect the output response (see Achieving biological signal differentiation). To this end, we consider the following, more complex, ODE model:

(Equation S84) $$\dot{X}=k_{in}U+b-k_{1}XZ_{1}-\left(\delta +\gamma \right)X$$

(Equation S85) $${\dot{Z}}_{1}=V_{max}\frac{X}{X+K_{m}}-\eta Z_{1}Z_{2}-\gamma Z_{1}$$

(Equation S86) $${\dot{Z}}_{2}=k_{3}-\eta Z_{1}Z_{2}-\gamma Z_{2}$$

where *γ* represents a dilution rate constant.

In general, linearization of Equations (S84), (S85), and (S86) around their steady-state (which is obviously different than before) results in a system which does not have the same form as Equation (S30) and, thus, the procedures of our original analysis are not valid here. Nevertheless, if the dilution effect is not strong, it can be seen from simulations that the behavior of this model approaches the one of Equations (S78), (S79), and (S80).

Note that the above structural ”perturbations” appear also in the natural systems discussed in Biomolecular signal differentiators in natural regulatory networks. In parallel, activation of species *X* by *Z* and $Z_{1}$ in BioSD-I and BioSD-III, respectively is also done through gene expression (see Guidelines for experimental implementation of biomolecular signal differentiators). In addition, dilution is present when realizing the latter topologies in living cells. Consequently, we can draw similar conclusions about them as with BioSD-II.

We now numerically investigate the behavior of BioSD-II. Figure S7A shows the response of the system given by Equations (S78), (S79), and (S80) to the input presented in Figure 3C using the parameter rates in Table S1, except for the dilution rate *γ* which is considered zero. As can be seen, BioSD-II can accurately calculate the rate of change of the input applied.

Note also the following:

• From Equation (S85) and Table S1 we calculate the steady-state concentration of species *X* which is equal to 20 nM. Based on the values of $V_{max}$, $K_{m}$ and taking into account that *X* moves around the aforementioned point, the production rate of species $Z_{1}$ can be approximated well by the term $k_{2}X$, where $k_{2}\approx 1$ (no saturation occurs).
• To facilitate the comparison of the BioSD output with the derivative of the input we choose a value for $k_{in}$ equal to the value of the quantity $k_{1}k_{3}$ (see Equation (S58)). At the same time, here input *U* represents an actuator species whose concentration is related linearly with the corresponding production rate of output species *X* (which may result from the linear regime of a Hill function as discussed above). Nevertheless, in the general case the term $k_{in}U$ can represent any (nonlinear) function describing the activating mechanism of the output species.
• From Equation (S53) we get ε≈0.125. Moreover, *η* can be considered sufficiently large since η=425 nM^−1^ min^−1^≫$\frac{\beta_{1}^{2}}{k_{3}}\approx 14.18$ nM^−1^ min^−1^ (see Equation (S36)). Consequently, Equation (S43) holds.
• Protein production rates regarding gene expression can be easily adjusted, for example, by changing gene copy number and, thus, a wide range of values can be achieved - a typical parameter range for *E*. *coli* is $0.5-10^{4}$ nM nM (Aoki et al., 2019). This implies extensive tunability which is important for meeting different performance standards (see Tunability and accuracy) since a considerable number of parameter rates in BioSD-II is associated with gene expression, i.e. *b*, $k_{2}$ (which is related to $V_{max}$, $K_{m}$), $k_{3}$ and $k_{in}$.

Figure S7B shows the response of the system given by (Equation S84), (Equation S85) and (S86) to the same input stimulus. We also use the same parameters rates as before except for the dilution rate which is now nonzero and equal to a typical value for *E*. *coli* (see Table S1). It is evident that the output remains an accurate replica of the derivative of the input.

Subsequently, in Figures S7C and S7D we further investigate the impact of dilution on the output of BioSD-II by repeating the simulation of Figure S7B with a 5 and 10 times larger dilution rate, respectively. We notice that as this rate gets stronger the actual response moves away from the zero-level “bias” which coincides with the corresponding output steady-state. Moreover, although the accuracy drops to some extent, the form of the output remains close to the one of the ideal derivative.

As already pointed out, the annihilation rate *η* is chosen to be sufficiently large so that the condition given by Equation (S43) is satisfied (only BioSD-II entails such a requirement). More specifically, *η* is approximately 30 times larger than the quantity $\frac{\beta_{1}^{2}}{k_{3}}$. Nevertheless, it remains unclear to us if such suitable values of *η* can be always guaranteed *in vitro* by the interaction between the pair of protease/protease inhibitor proposed in Guidelines for experimental implementation of biomolecular signal differentiators. It is therefore important to investigate the behavior of the differentiator module in the case where *η* is not as large as our theoretical analysis demands. As shown in Figure S8, non-satisfaction of the condition given by Equation (S43) does not necessarily entail significant loss of accuracy regarding signal differentiation. Note also that the quantity $\frac{\beta_{1}^{2}}{k_{3}}$ can be easily adjusted to a suitable value by appropriately tuning the protein production rates involved in BioSD-II (discussed earlier).

Finally, to make the above analysis even more realistic (Del Vecchio and Murray, 2015), one could model gene expression as a multi-stage process, thus capturing the dynamics of transcription and translation. At the same time, the dynamics of complexes participating in intermediate stages of inhibition and annihilation reactions could also be considered. Nonetheless, it is important to emphasize that such an approach would increase the complexity of the resulting mathematical models.

## Data Availability

All numerical simulations were performed in MATLAB R2020 using the ODE solver ode23s except for those in Figure 5 where the ODE solver ode113 was used. Simulation parameter values can be found in the figure captions. Initial conditions for the biomolecular species involved are considered zero except for BioSDs and BioSDs^*F*^ where the corresponding equilibria (“rest-positions”) are used (see STAR Methods Equilibria and stability of Biomolecular Signal Differentiators and An alternative version of Biomolecular Signal Differentiators). The corresponding programming code is available at: https://github.com/emgalox/BioS-Differentiators.
