# Supplementary material for: Biomolecular mechanisms for signal differentiation
Source: iScience. 2021 Nov 17;24(12):103462. doi: 10.1016/j.isci.2021.103462 (PMC8649740; doi:10.1016/j.isci.2021.103462)
Supplement: Document S1. Figures S1–S8 and Table S1 [file mmc1.pdf]

iScience, Volume *24*

Supplemental information

Biomolecular mechanisms for signal differentiation

Emmanouil Alexis, Carolin C.M. Schulte, Luca Cardelli, and Antonis Papachristodoulou

	Description	Value	Unit	Comments	Source
γ	Dilution rate	0.028	min^{-1}	Value for *E. coli*, assuming 25 min doubling time	Aoki et al., 2019
δ	Degradation rate	0.1	min^{-1}	Unspecified mechanism (disturbance) contributing to degradation	Aoki et al., 2019
η	Annihilation rate	425	nM^{-1} min^{-1}	Value based on binding rates for protein-protein interactions that are diffusion-limited	Schlosshauer and Baker, 2004; Fekkes, Blaauwen, and Driessen, 1995
k_1	Catalytic inhibition rate	1.6	nM^{-1} min^{-1}	Value based on the action of Lon protease	Gur, Vishkautzan, and Sauer, 2012
k_3	Constitutive production rate	20	nM min^{-1}		Buchler and Louis, 2008; Aoki et al., 2019
b	Constitutive production rate	40	nM min^{-1}		Buchler and Louis, 2008; Aoki et al., 2019
V_{max}	Maximal production rate	900	nM		Buchler and Louis, 2008; Aoki et al., 2019
K_m	Michaelis-Menten constant	880	nM		Buchler and Louis, 2008; Aoki et al., 2019

Table S1: Simulation parameters for STAR Methods **Modelling a more realistic case of Biomolecular Signal Differentiator-II**

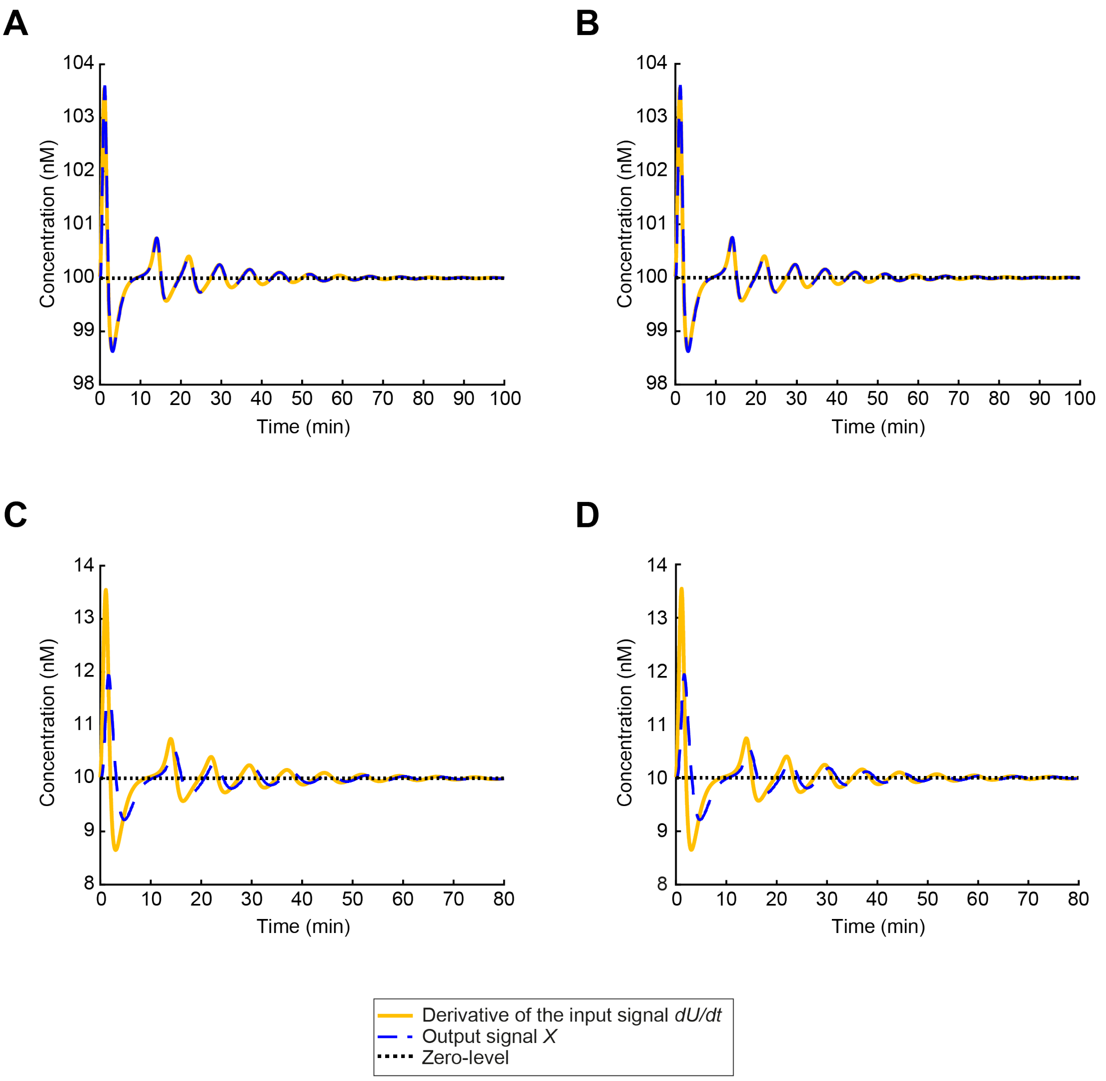

Figure S1: Sensing the rate-of-change of a synthetic regulatory biomolecular network through a Biomolecular Signal Differentiator. Related to Figure 3.
a Simulation of the BioSD-II (Equations (S11)-(S13)) response to the input presented in Figure 3c with $\eta = 3000$ nM^{-1} min^{-1}, $b = 150$ nM min^{-1} and the remaining parameters same as those used in Figure 3d. η can be characterized as sufficiently large since condition (S43) is satisfied. **b** Simulation of the BioSD-III (Equations (S23)-(S25)) response to the input presented in Figure 3c with $\eta = 30$ nM^{-1} min^{-1} and the remaining parameters same as those used in Figure 3d. **c** The simulation in **a** is repeated with the values of k_{in}, k_3, b set to 10, 10 and 100, respectively. **d** The simulation in **b** is repeated with the values of both k_{in} and k_3 set to 10. As can be seen, the behaviour of both BioSD-II and BioSD-III is identical to that of BioSD-I depicted in the main text. As a result, the conclusions drawn with respect to the latter circuit are valid for the other designs as well.

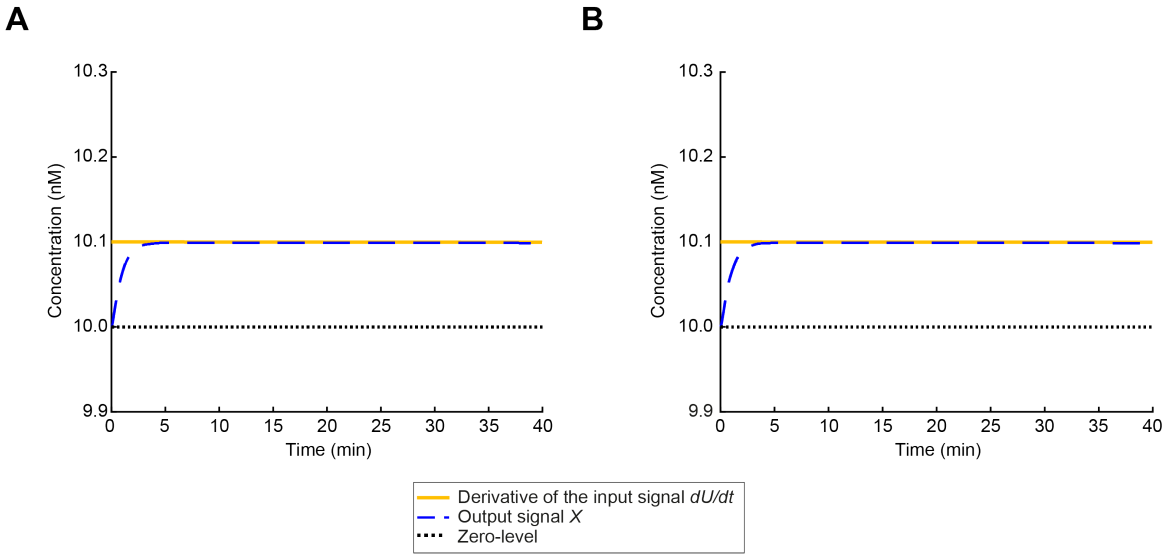

Figure S2: Sensing the rate-of-change of a birth-death biomolecular process through a Biomolecular Signal Differentiator. Related to Figure 4.
a Simulation of the BioSD-II (Equations (S11)-(S13)) response to the input presented in Figure 4c with $\eta = 3000$ nM^{-1} min^{-1} and the remaining parameter same as those used in Figure 4d. η can be described as sufficiently large since condition (S43) is satisfied. **b** Simulation of the BioSD-III (Equations (S23)-(S25)) response to the input presented in Figure 4c with $\eta = 30$ nM^{-1} min^{-1} and the remaining parameter same as those used in Figure 4d. As can be seen, the behaviour of both BioSD-II and BioSD-III is identical to that of BioSD-I depicted in the main text. As a result, the conclusions drawn with respect to the latter circuit are valid for the other designs as well.

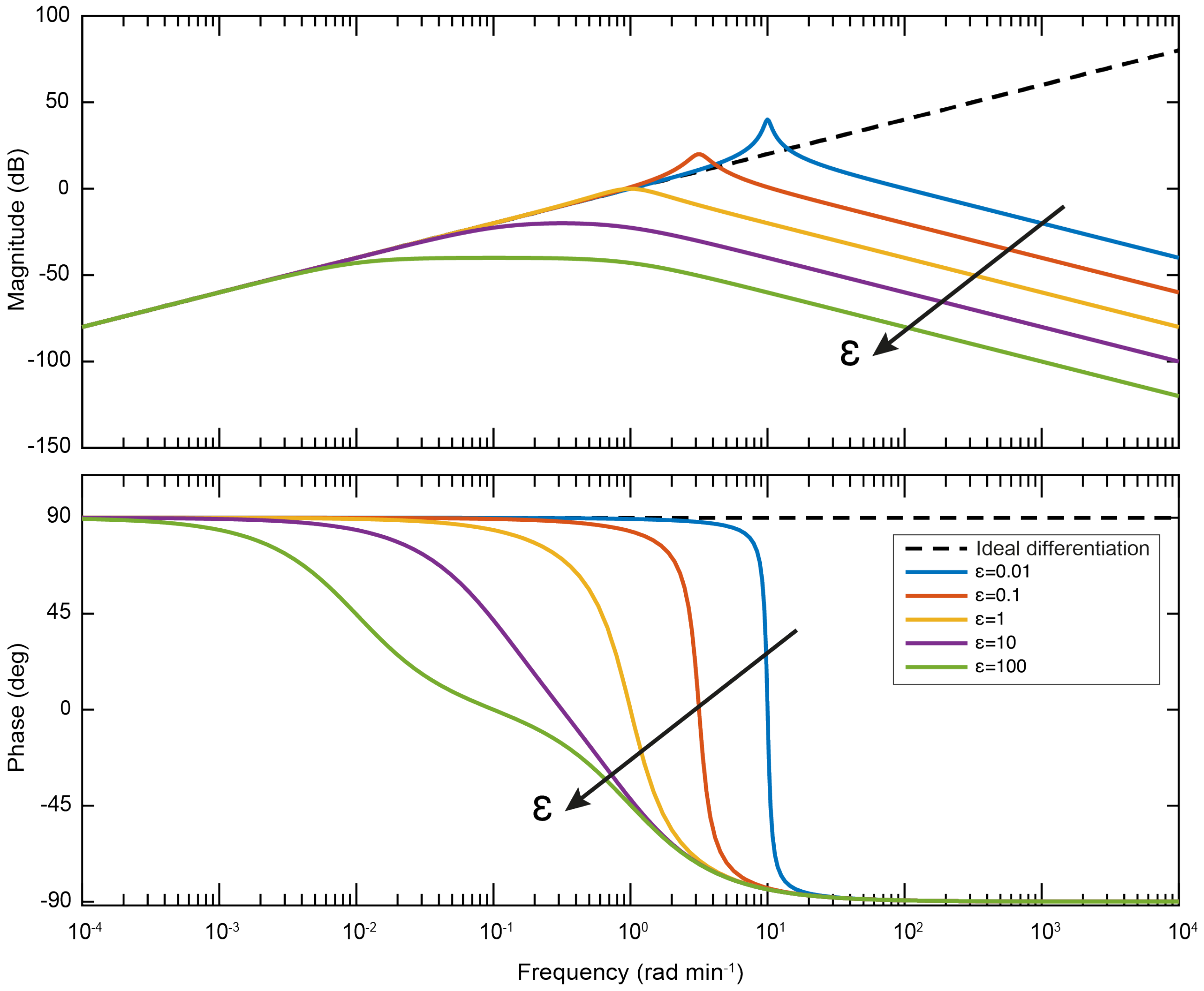

Figure S3: Frequency response analysis of Biomolecular Signal Differentiators. Related to STAR Methods. Bode plot of a BioSD differentiator (Equation (S62)). The magnitude and the phase of its transfer function are depicted for different values of ε via distinct colours.The case of ideal differentiation corresponds to $\varepsilon = 0$ and the direction in which the latter increases indicated by an arrow..

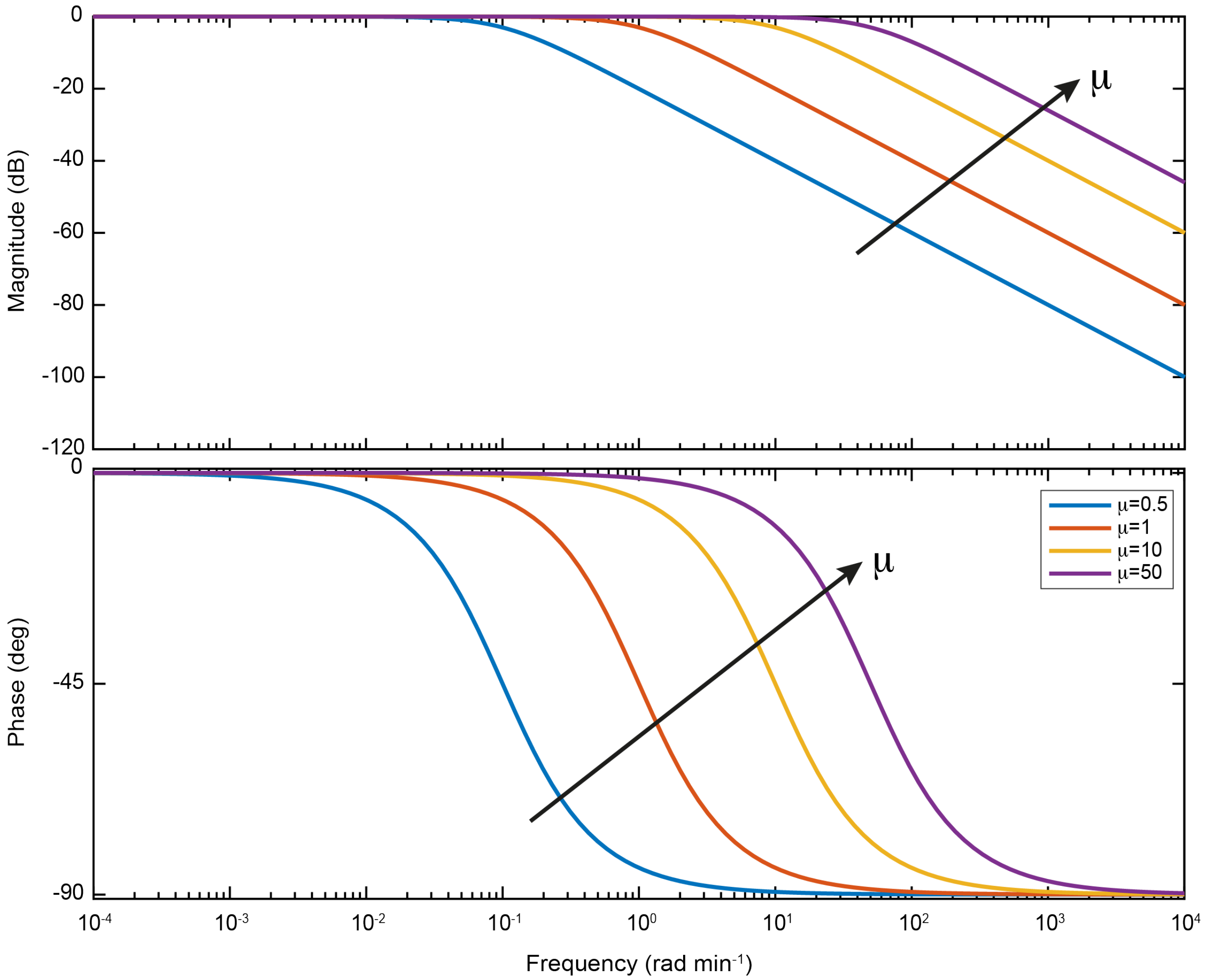

Figure S4: Frequency response analysis of the subsystem that receives the input signal U. Related to STAR Methods.

Bode diagram of the filter module described by Equation (S69). The magnitude and and the phase lag of its frequency response for different values of μ are shown in different colours where $\mu = \mu_1 = \mu_2$. The increasing direction of μ indicated by an arrow.

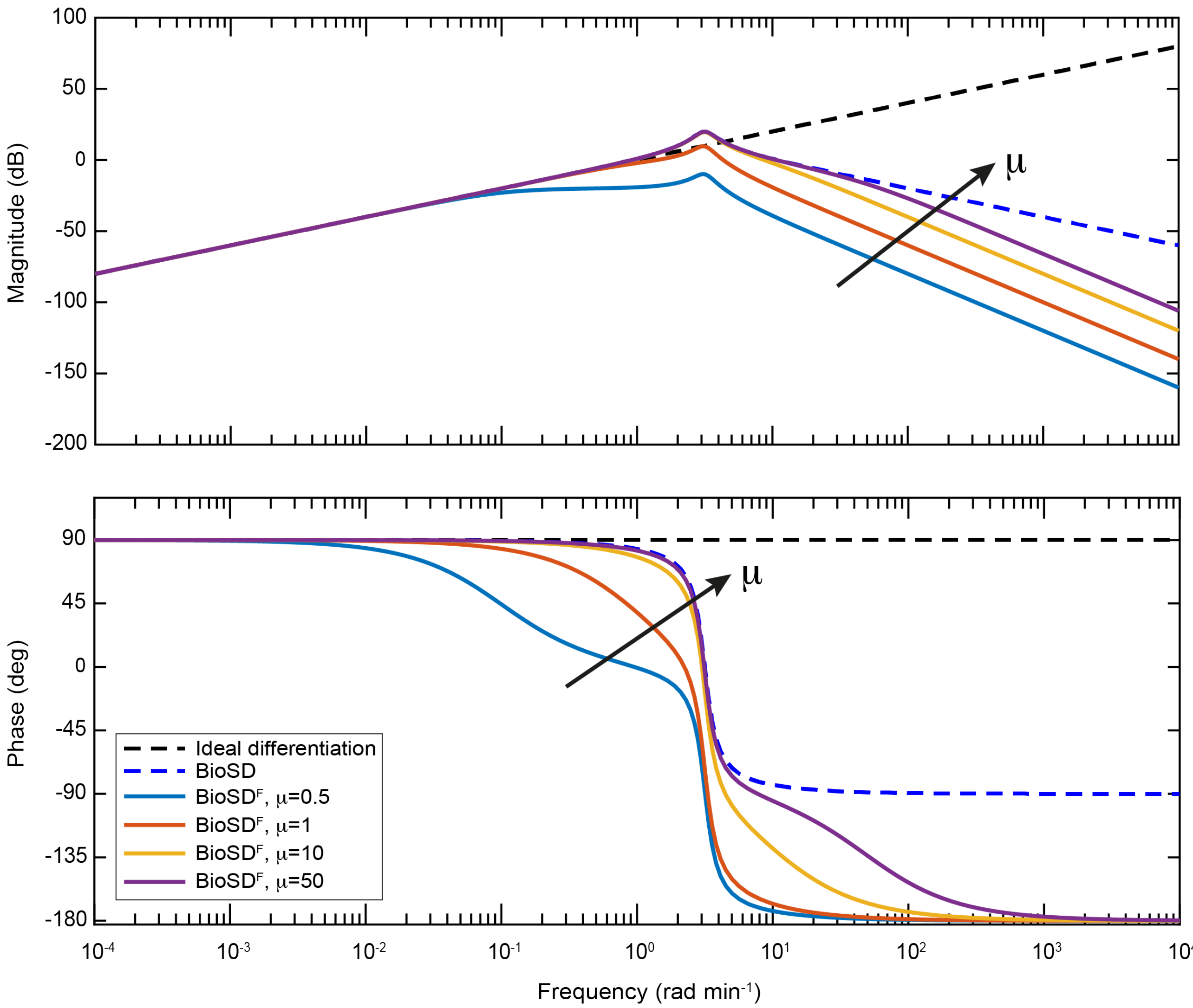

Figure S5: Frequency response analysis of Biomolecular Signal Differentiators^F. Related to STAR Methods. Bode diagram depicting the magnitude and phase shift regarding the frequency response of a BioSD^F differentiator (Equation (S71)) with $\varepsilon = 0.1$. We consider different values of μ, where $\mu = \mu_1 = \mu_2$, that correspond to solid lines of different colours while the increasing direction of μ indicated by an arrow. For comparison purposes, we also depict the Bode plot (magnitude and phase) of a BioSD differentiator (Equation (S62)) with $\varepsilon = 0.1$ and the one of an ideal differentiator which are represented by blue and black dashed lines, respectively.

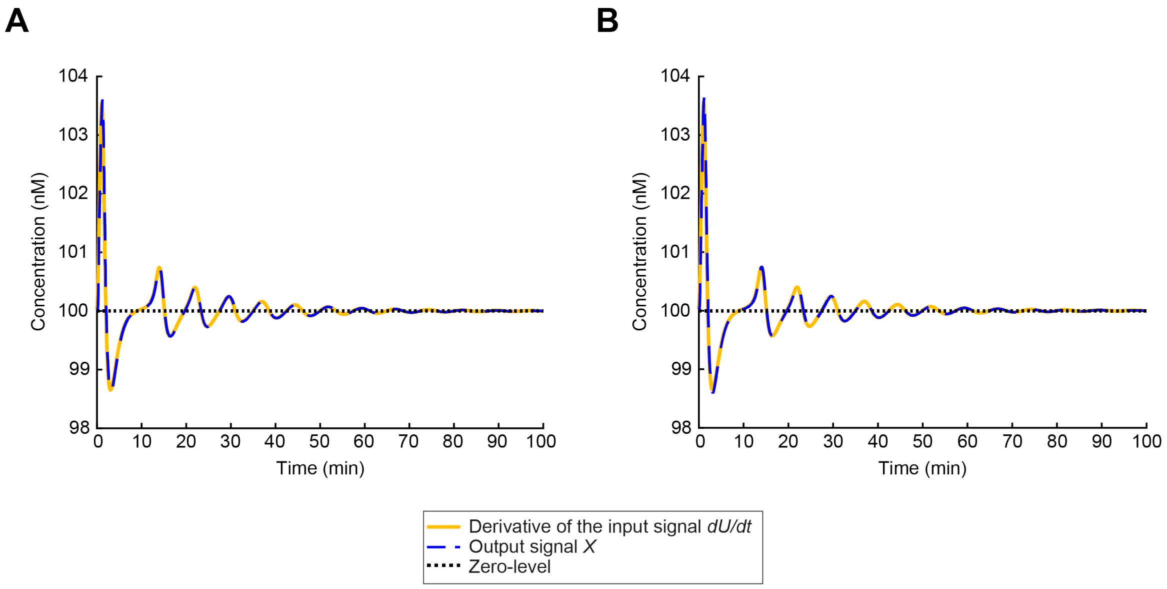

Figure S6: Sensing the rate-of-change of a synthetic regulatory biomolecular network through the proposed (experimental) circuit of Biomolecular Signal Differentiator-III. Related to STAR Methods.
a Simulation of the circuit given by Equations (S72)-(S77) using the input presented in Figure 3c and the following parameters: $k_{in} = 100$ min^{-1}, $k_3 = b = 100$ nM min^{-1}, $k_1 = k_{1a} = k_{1b} = 1$ nM^{-1} min^{-1}, $k_2 = 1$ min^{-1}, $\eta = \eta_a = 30$ nM^{-1} min^{-1}, $\delta = \delta_a = 0.5$ min^{-1} (this scenario corresponds to the simulation depicted in Figure S1b). **b** We repeat the simulation in **a** with the values of k_{1a}, k_{1b}, η_a, δ_a set to 1.5 (increase by 50%), 1.25 (increase by 25%), 45 (increase by 50%), 0.75 (increase by 50%), respectively.
It is evident that in both **a** (ideal case) and **b** the output, X, of the differentiator is an accurate replica of the derivative of input U - the loss of accuracy in **b** is negligible.

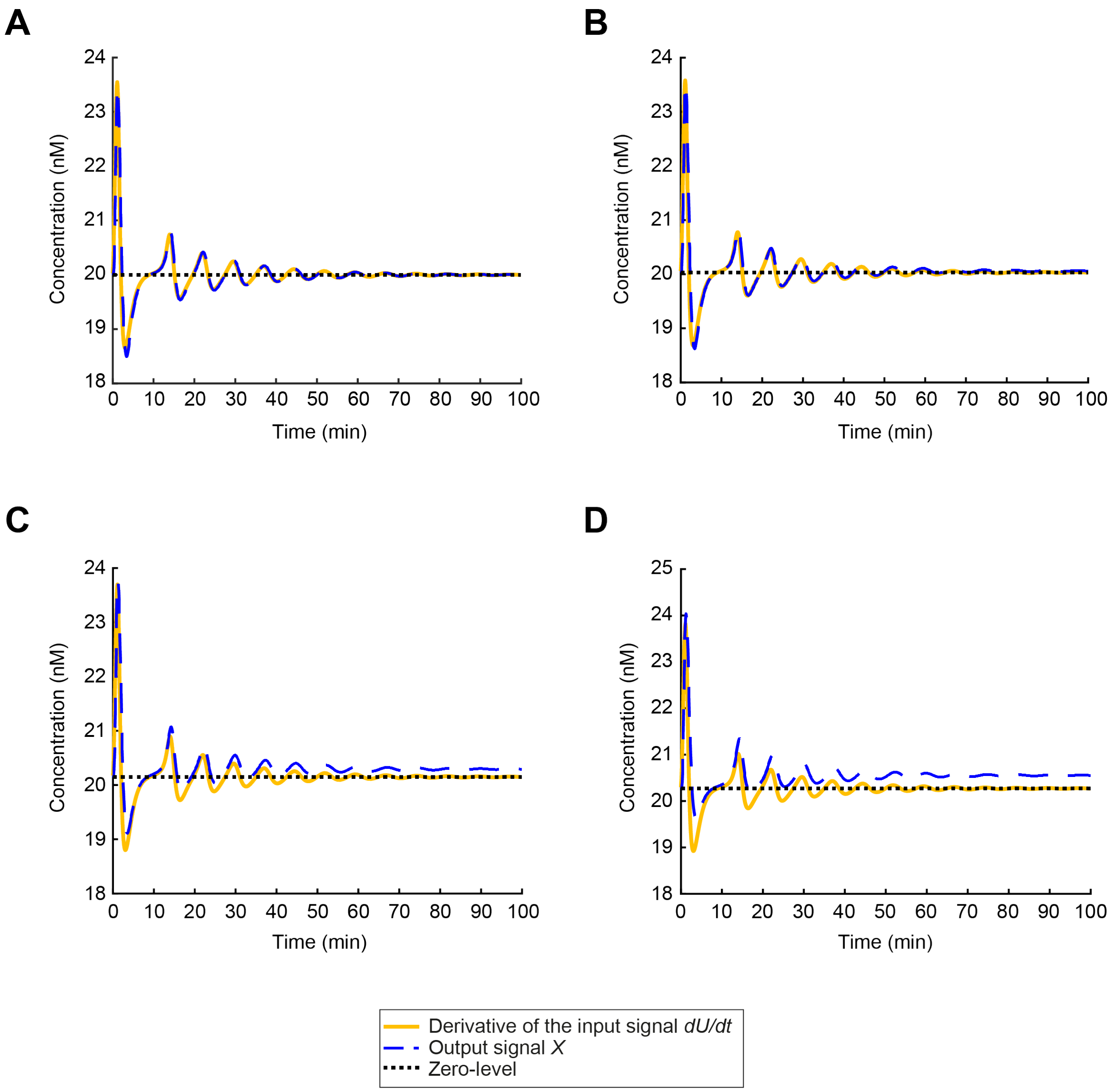

Figure S7: Sensing the rate-of-change of a synthetic regulatory biomolecular network through a more realistic model of Biomolecular Signal Differentiator-II. Related to STAR Methods.

a Simulation of the system given by Equations (S78)-(S80) using the input presented in Figure 3c and the parameters of Table S1 (no dilution). **b** Simulation of the system given by Equations (S84)-(S86) using the input presented in Figure 3c and the parameters of Table S1. **c** The simulation in **b** is repeated with a five times larger dilution rate, i.e. $\gamma = 0.14$ min^{-1}. **d** The simulation in **b** is repeated with a ten times larger dilution rate, i.e. $\gamma = 0.28$ min^{-1}.

In all the simulations we assume that the value of k_{in} is equal to the value of the quantity $k_1 k_3$.

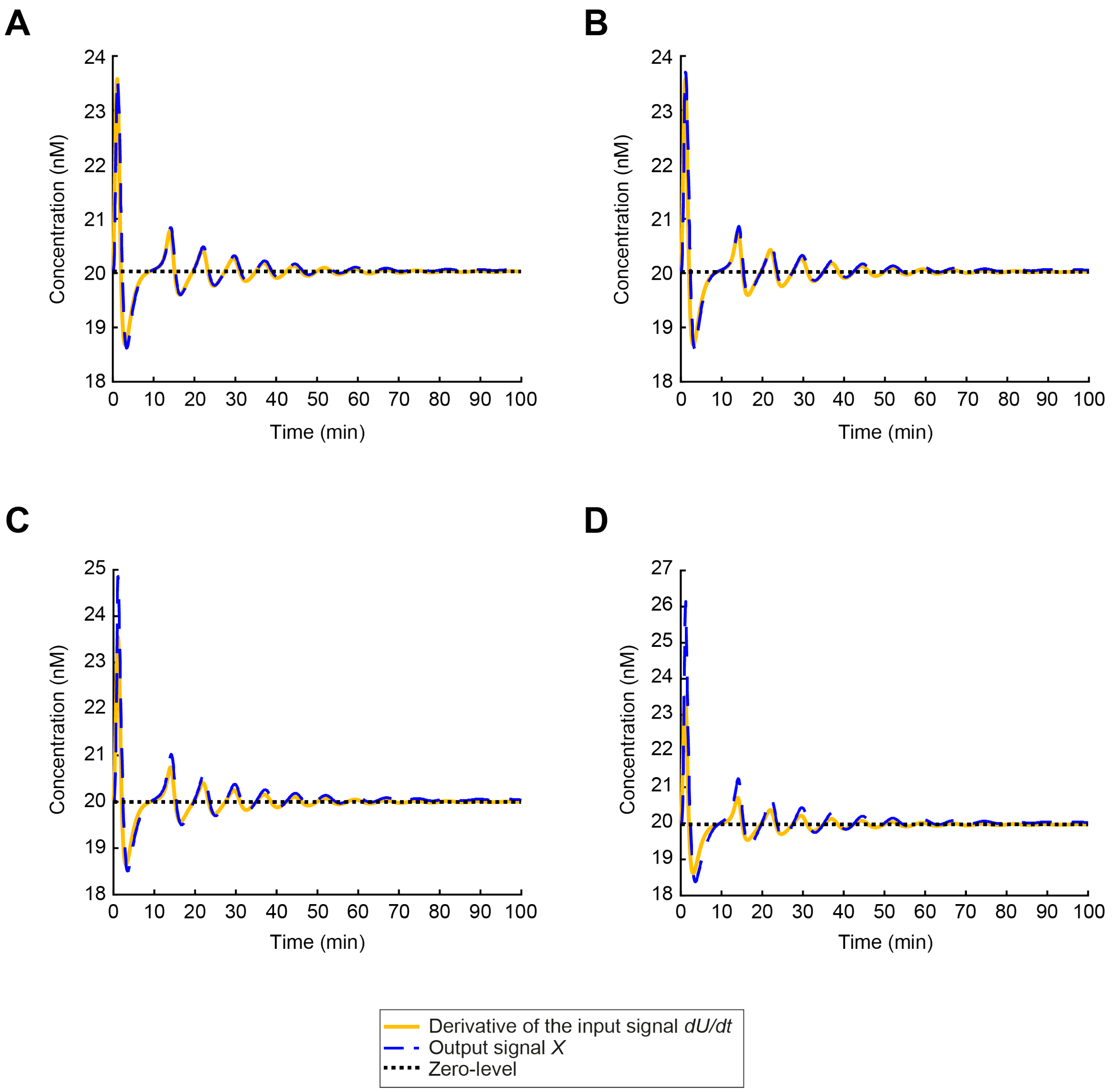

Figure S8: Sensing the rate-of-change of a synthetic regulatory biomolecular network through a more realistic model of Biomolecular Signal Differentiator-II with a lower annihilation rate, η, than the one of Table S1. Related to STAR Methods.

Simulation of the system given by Equations (S84)-(S86) using the input presented in Figure 3c and **a** $\eta = 10\frac{\beta_1^2}{k_3}$, **b** $\eta = 5\frac{\beta_1^2}{k_3}$, **c** $\eta = \frac{\beta_1^2}{k_3}$, **d** $\eta = 0.5\frac{\beta_1^2}{k_3}$ which correspond to 141.8 nM^{-1} min^{-1}, 70.9 nM^{-1} min^{-1}, 14.18 nM^{-1} min^{-1} and 7.09 nM^{-1} min^{-1}, respectively (see STAR Methods **Modelling a more realistic case of Biomolecular Signal Differentiator-II**). In addition, the value of k_{in} is assumed to be equal to the value of the quantity $k_1 k_3$ while rest of the parameters are in accordance with Table S1.
